# Novel 2,3-Dihydro-1*H*-pyrrolo[3,2,1-*ij*]quinazolin-1-ones: Synthesis and Biological Evaluation

**DOI:** 10.3390/molecules22010055

**Published:** 2016-12-30

**Authors:** Malose J. Mphahlele, Tebogo A. Khoza, Peaceful Mabeta

**Affiliations:** 1 Department of Chemistry, College of Science, Engineering and Technology, University of South Africa, Private Bag X06, Florida 1710, South Africa; khozata@unisa.ac.za; 2 Department of Anatomy and Physiology, University of Pretoria, P/Bag X04, Pretoria 0110, South Africa; Peace.Mabeta@up.ac.za

**Keywords:** 1*H*-pyrrolo[3,2,1-*ij*]quinazolin-1-ones, X-ray, cytotoxicity, *Plasmodium falciparum*, antiplasmodial activity

## Abstract

Herein we describe the synthesis and evaluation of a series of novel 2,3-dihydro-1*H*-pyrrolo[3,2,1-*ij*]quinazolin-1-ones for in vitro cytotoxicity against three human cancer cell lines as well as for potential antimalarial activity against the chloroquine-sensitive strain 3D7 of *Plasmodium falciparum*. The title compounds were prepared via PdCl_2_-mediated *endo-dig* cyclization of 2-aryl-8-(arylethynyl)-6-bromo-2,3-dihydroquinazolin-4(1*H*)-ones. The latter were prepared, in turn, via initial Sonogashira cross-coupling of 2-amino-5-bromo-3-iodobenzamide with aryl acetylenes followed by boric acid-mediated cyclocondensation of the intermediate 2-amino-3-(arylethynyl)-5-bromobenzamides with benzaldehyde derivatives. The 2,3-dihydro-1*H*-pyrrolo[3,2,1-*ij*]quinazolin-1-ones **4a**–**k** were evaluated for potential in vitro cytotoxicity against the breast (MCF-7), melanoma (B16) and endothelioma (sEnd.2) cell lines. All of the compounds except **4h** and **4i** were found to be inactive against the three cancer cell lines. Compound **4h** substituted with a 4-methoxyphenyl and 4-fluorophenyl groups at the 3- and 5-positions was found to exhibit significant cytotoxicity against the three cancer cell lines. The presence of phenyl and 3-chlorophenyl groups at the 3- and 5-posiitons of the pyrroloquinazolinone **4i**, on the other hand, resulted in significant cytotoxicity against vascular tumour endothelial cells (sEnd.2), but reduced activity against the melanoma (B16) and breast cancer (MCF-7) cells except at higher concentrations. The 2,3-dihydro-1*H*-pyrrolo[3,2,1-*ij*]quinazolin-1-ones **4a**–**l** were found to be inactive against the chloroquine sensitive 3D7 strain of *Plasmodium falciparum*.

## 1. Introduction

**Figure 1:**
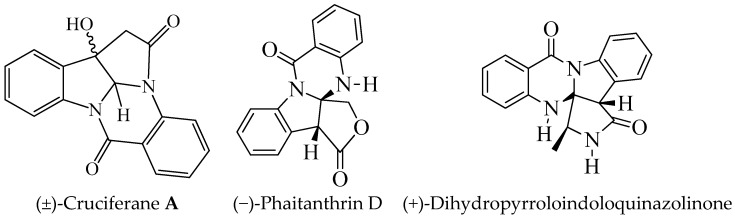
Structures of the racemic pyrrolo[2,3-*b*]indolo[5,5-*a*,6-*b*,*a*]quinazolinone (criciferane **A**); (−)-phaitanthrin D and (+)-dihydropyrroloindoloquinazolinone.

The design and synthesis of pyrroloquinazolinone-based compounds continue to attract considerable attention in synthetic organic chemistry due to their medicinal applications [1,2,3,4]. The 3-alkyl substituted 7-phenyl-3*H*-pyrrolo[3,2-*f*]quinazolin-9-ones and the 2,7-dihydro-1*H*-pyrrolo[3,2-*f*]quinazolin-1-ones, for example, have been found to exhibit significant in vitro inhibitory activity in the micromolar range against tubulin polymerization and thrombin-induced aggregation, respectively [1]. Both quinazolin-4(1*H*)-one and indole moieties are an integral part of the naturally occurring racemic pyrrolo[2,3-*b*]indolo[5,5-*a*,6-*b*,*a*]quinazolinone [(±)-cruciferane] **A** (Figure 1), which was first isolated from the roots of *Isatis indigotic* [2,5]. (−)-Phaitanthrin D and (+)-dihydropyrroloindoloquinazolinone depicted in Figure 1, on the other hand, were recently prepared from enantiomerically pure anthranilamide-based building blocks using HMDS/ZnCl_2_ and NaHMDS as reagents [3]. The most common methods for the synthesis of pyrroloquinazolinones generally make use of the 5-amino–substituted indoles as substrates for condensation with ethyl benzoylacetate [1,5] or *N-*ethoxycarbonylthiobenzamides [1,4] followed by thermal cyclization of the incipient ethyl 3-indoleamino-3-phenylacrylates or *N-*indole-*N*′-ethoxycarbonylamidine, respectively. Several naturally occurring and synthetic 2,3-dihydroquinazolin-4(1*H*)-ones have themselves been found to exhibit interesting biological properties such anti-inflammatory, anticancer, anticonvulsant and anti-hypertensive activities [6,7,8]. Likewise, indole-based compounds also exhibit pharmacological properties such as antimicrobial, antimalarial, antioxidant and anti-tubercular activities, and this moiety is also widely distributed in numerous natural products [9,10].

Despite growing interest in the synthesis of pyrroloquinazolinones [4], an extensive literature search revealed that molecular hybridization to integrate indole and dihydroquinazolin-1*H*-one moieties to generate the 1*H-*pyrrolo[3,2,1-*ij*]quinazolin-1-ones has not been explored. Moreover, these angularly fused ring systems have not been described in recent reviews on synthetic approaches, functionalization and therapeutic potential of quinazoline and quinazolinone skeletons [2,7,11]. We envisioned that molecular hybridization to construct a pyrrole ring onto the dihydroquinazolinone framework through the standard indole synthesis would lead to the 2,3-dihydro-1*H*-pyrrolo[3,2,1-*ij*]quinazolin-1-ones. In our view, 2-amino-5-bromo-3-iodobenzamide [12,13] represented a suitable substrate for the initial Sonogashira cross-coupling with arylalkynes followed by cyclocondensation of the intermediate 2-amino-3-(arylalkynyl)benzamides with benzaldehyde derivatives to afford the corresponding 8-alkynylated 2,3-dihydroquinazolin-4(1*H*)-ones. The latter would undergo transition metal-assisted *endo-dig* C*sp*–N cyclization to afford the required 2,3-dihydro-1*H*-pyrrolo[3,2,1-*ij*]quinazolin-1-ones. The prepared compounds would, in turn, be evaluated for in vitro cytotoxicity and antimalarial properties.

## 2. Results and Discussion

### 2.1. Chemistry

**Scheme 1:**
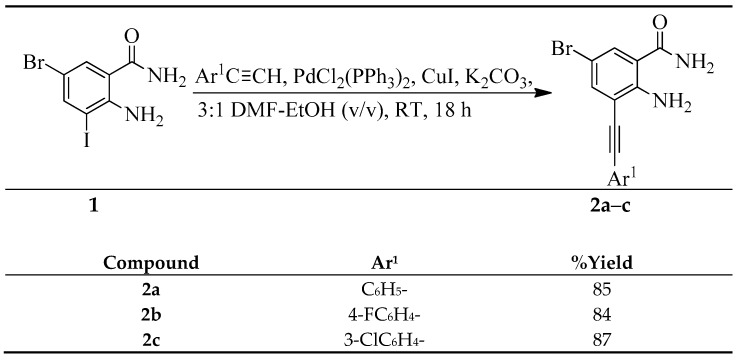
Site selective C-3 alkynylation of **1**.

We took advantage of the more reactive C*sp*^2^–iodine bond to undergo transition metal catalysed oxidative-addition with ease in the presence of C*sp*^2^–Br bond and subjected 2-amino-5-bromo-3-iodobenzamide **1** to the Sonogashira cross-coupling with aryl acetylenes (1 equivalent) in the presence of PdCl_2_(PPh_3_)_2_-CuI catalyst mixture and K_2_CO_3_ as a base in 3:1 DMF-ethanol (*v*/*v*) at room temperature (RT) for 18 h (Scheme 1). We isolated in each case by column chromatography on silica gel traces of the homo-coupled dimer and a product characterised using a combination of NMR and IR spectrometric techniques as the 3-alkynylated benzamide **2** (Scheme 1). The calculated *m*/*z* values for the cross-coupled products were found to be consistent with the molecular ions of the assigned structures.

**Scheme 2:**
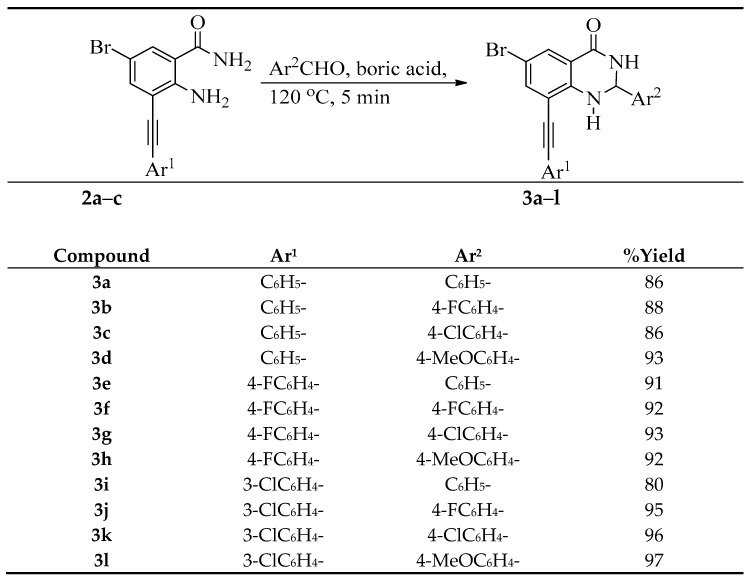
Boric acid-mediated cyclocondensation of compounds **2a**–**c** with the benzaldehyde derivatives.

Our next focus was to subject compounds **2a**–**c** to cyclocondensation reactions with benzaldehyde derivatives. We followed a method described in the literature [14], which involves heating a mixture of the 2-aminobenzamide derivative and the aryl aldehyde in the presence of boric acid at 120 °C, followed by an aqueous work-up. Thus, mixtures of **2a**–**c** and the benzaldehyde derivatives as well as boric acid (20 mol% relative to **2**) were finely ground in crucibles and then transferred to round-bottomed flasks. This was followed by heating at 120 °C for 5 min and the solidified crude mixtures were washed thoroughly with water and then recrystallized from ethanol to afford the corresponding 8-arylalkynyl substituted 2,3-dihydroquinazolin-4(1*H*)-ones **3a**–**l** in excellent yields (Scheme 2).

**Scheme 3:**
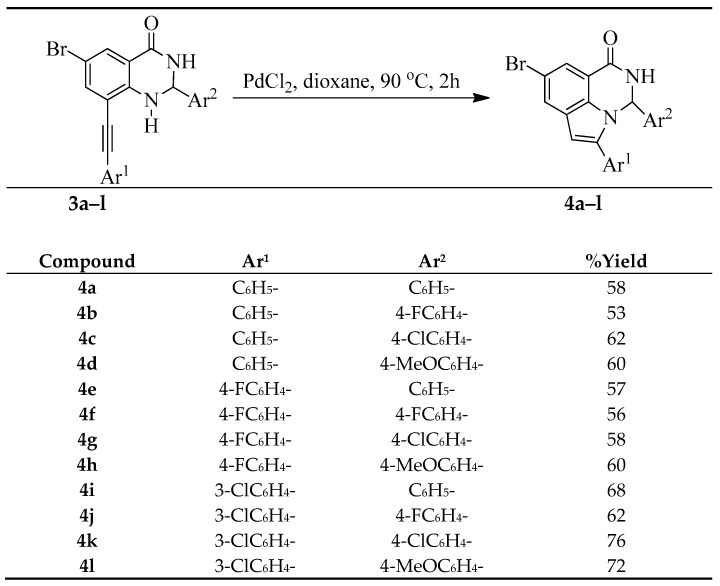
PdCl_2_-mediated *endo-dig* cyclization of **3a**–**l**.

The cyclization of alkynylated heteroatom-containing compounds in which the alkynyl group is located adjacent to a nucleophilic heteroatom (N, O, S) represents a very effective strategy for the Lewis acid or transition metal mediated cyclization to afford heterocyclic derivatives [15]. We became interested by this approach and attempted to effect iodine-mediated cyclization of compound **3a** in the presence of molecular iodine (I_2_) in methanol under reflux and also in the presence of a mixture of iodine and sodium carbonate in dichloromethane or tetrahydrofuran first at RT and then under reflux. In both cases, we recovered the starting material without traces of the expected product detected in the mixture. We then followed another literature procedure, which involves the use of palladium chloride (PdCl_2_) in acetonitrile (CH_3_CN) under reflux. These reaction conditions have previously been employed on the 8-alkynylated 2,3-dihydroquinolin-4(1*H*)-ones to afford the corresponding 2,3-dihydro-1*H*-pyrrolo[3,2,1-*ij*]quinolin-1-ones [16]. Since compounds **3a**–**l** were found to be insoluble in acetonitrile, we reacted them with PdCl_2_ in dioxane as a solvent at 100 °C for 2 h (Scheme 3). After aqueous work-up and purification by silica gel chromatography we isolated the 2,3-dihydro-1*H*-pyrrolo[3,2,1-*ij*]quinazolin-1-ones **4a**–**l** as products and these were confirmed by NMR and IR spectrometric techniques.

**Figure 2:**
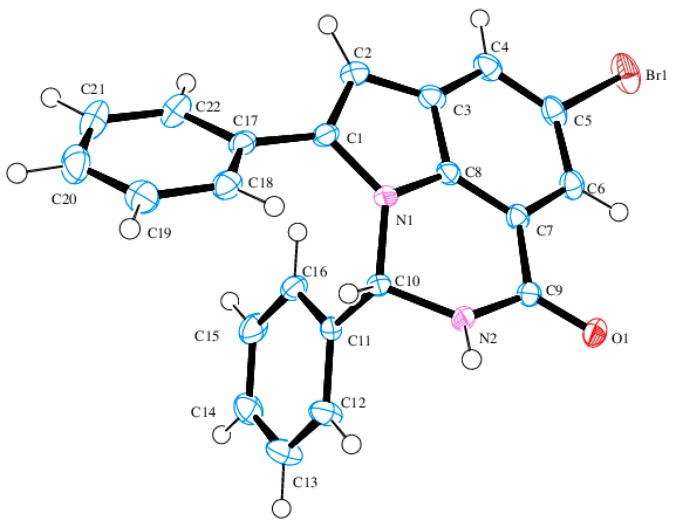
Oak Ridge Thermal Ellipsoid Plot (ORTEP) diagram (50% probability level) of **4a** showing crystallographic numbering.

We obtained crystals of suitable quality for X-ray diffraction studies for compound **4a**. The polynuclear structure of compounds **4** was confirmed independently by single crystal X-ray diffraction [17]. The 3-phenyl and 5-phenyl rings of compound **4a** are twisted out of the plane of the heterocyclic framework with average torsion angels of −127.5° and −148.6°, respectively. The crystal structure shows the presence of strong intermolecular hydrogen bonding between N-H of one molecule and the carbonyl oxygen of another with D–H^…^A angle of 156.4° and H^…^A distance of 2.00 Å (Figure 2).

Pyrroloquinazolines have been found to exhibit a wide range of pharmacological activities and some serve as inhibitors of dihydrofolate reductase (DHFR) and protein tyrosine phosphatase, antimicrobial agents, protease activated receptor antagonists and thrombin receptor antagonists [2,18]. These literature precedents encouraged us to evaluate compounds **4** for potential biological properties. We decided to screen compounds **4a**–**l** for potential in vitro cytotoxicity against three cancer cell lines, namely, breast, melanoma and endothelioma cells. We also evaluated these compounds for potential antimalarial activity against the chloroquine-sensitive strain 3D7 of *Plasmodium falciparum* as described in the next section.

### 2.2. Biology

#### 2.2.1. In Vitro Cytotoxicity Studies of the Pyrrolo[3,2,1-*ij*]quinazolin-1-ones **4**

Compounds **4a**–**k** were evaluated for in vitro cytotoxicity against the breast cancer (MCF-7), melanoma (B16) and endothelioma (sEnd.2) cell lines using the crystal violet nuclear staining assay. The compounds were assayed in triplicate at concentrations ranging from 0 to 50 μM with 0.01% DMSO as the negative control. DMSO at 1% or less has been reported to have no effect on proliferation of the HeLa and the Caco_2_ cells for up to 48 h [19,20]. The IC_50_ values (the concentration of compound that reduced cell viability by half) for compounds **4a**–**k** (average from three independent experiments) are represented in Table 1 in μM concentrations, taking into account the molecular weights of the compounds (see Supplementary Materials for the corresponding cell viability percentages and graphs for each compound).

Only compounds **4h** and **4i** were found to exhibit significant in vitro cytotoxicity. Compound **4h** substituted with 4-methoxyphenyl and 4-fluorophenyl at the 3- and 5-position was found to be the most potent of all compounds tested with IC_50_ values of 0.83, 0.66 and 0.95 μM against the MCF-7, B16 and sEnd.2 cells, respectively. Compound **4i**, on the other hand, was found to exhibit significant in vitro cytotoxicity and selectivity against the vascular tumor endothelial cells with an IC_50_ value of 0.80 μM. This compound was found to exhibit moderate activity against the melanoma and breast cancer cells with IC_50_ values 9.36 μM and 11.35 μM, respectively. It seems the presence of a phenyl ring (see **4a**, **4e** and **4i**) or a 4-methoxyphenyl ring (see **4d** and **4h**) at the 3-position of a 1*H*-pyrrolo[3,2,1-*ij*]quinazolin-1-one framework is preferred over the 4-halogenophenyl substituent. All the compounds substituted with a 4-halogenophenyl group at the 3-position were found to lack of activity against the three cancer cell lines. The 2,3-dihydro-1*H*-pyrrolo[3,2,1-*ij*]quinazolin-1-ones **4a**–**l** were also evaluated for potential in vitro antiplasmodial activity as described below.

#### 2.2.2. In Vitro Antiplasmodial Activity Studies of the Pyrrolo[3,2,1-*ij*]quinazolin-1-ones **4a**–**l**

The pyrrolo[3,2,1-*ij*]quinazolin-1-ones **4a**–**l** were evaluated for potential in vitro antimalarial activity against the chloroquine-sensitive 3D7 strain of *Plasmodium falciparum* using parasite lactate dehydrogenase (pLDH) assay [21]. The compounds were assayed in triplicate at concentrations ranging from 5.13–100 nM with DMSO and chloroquine (0.05–11,000 nM) as the negative and positive controls, respectively (see Supplementary Materials for the corresponding parasite survival percentages and graphs for each compound). The preliminary results revealed that compounds **4a**–**l** are generally inactive against the chloroquine-sensitive strain 3D7 of *P. falciparum* with IC_50_ values > 10 μM (Table 2). Based on this observation, we concluded that the 2,3-dihydro-1*H-*pyrrolo[3,2,1-*ij*]quinazolin-1-one moiety does not represent a suitable template for the development of compounds with antimalarial properties.

## 3. Experimental Section

### 3.1. General Information

Melting points were recorded on a Thermocouple digital melting point apparatus (Stuart, Staffordshire, UK) and are uncorrected. IR spectra were recorded as powders using a Bruker VERTEX 70 FT-IR Spectrometer (Bruker Optics, Billerica, MA, USA) with a diamond ATR (attenuated total reflectance) accessory by using the thin-film method. For column chromatography, kieselgel 60 (0.063–0.200 mm) (Merck KGaA, Frankfurt, Germany) was used as the stationary phase. NMR spectra were obtained as DMSO-*d*_6_ solutions using Varian 300 MHz (Varian Inc., Palo Alto, CA, USA) or Agilent 500 MHz NMR (Agilent Technologies, Oxford, UK) spectrometers and the chemical shifts were quoted relative to the TMS peak. Low- and high-resolution mass spectra were recorded using a Waters Synapt G2 Quadrupole Time-of-flight mass spectrometer (Waters Corp., Milford, MA, USA) at the University of Stellenbosch Mass Spectrometry Unit. The synthesis and analytical data of compound **1** have been described previously [13].

### 3.2. Typical Procedure for the Sonogashira Cross-Coupling of ***1***

A stirred mixture of **1** (1.00 g, 2.94 mmol), PdCl_2_(PPh_3_)_2_ (0.10 g, 0.15 mmol), CuI (0.06 g; 0.29 mmol) and K_2_CO_3_ (0.14 g, 1.66 mmol) in 3:1 DMF–EtOH (*v*/*v*, 20 mL) was purged with argon gas for 30 min. Phenyl acetylene (0.33 g, 3.22 mmol) was added via a syringe and the reaction mixture was stirred at RT for 18 h. The mixture was then quenched with ice-cold water and the product was extracted into chloroform. The combined organic layers were washed with water, dried over Na_2_SO_4_, filtered and then evaporated under reduced pressure. The residue was purified by column chromatography on silica gel to afford **2a**. The following products were prepared in this fashion.

*2-Amino-5-bromo-3-(phenylethynyl)benzamide* (**2a**). Solid (1.00 g, 85%), *R*_f_ (7:3 hexane–EtOAc) 0.60, m.p. 181–182 °C; IR (ATR): 534, 632, 667, 758, 1239, 1399, 1545, 1594, 1647, 3171, 3368, 2477 cm^−1^; ^1^H-NMR (500 MHz, DMSO-*d*_6_) 5.68 (2H, br s, NH_2_), 6.46 (2H, s, -CONH_2_), 7.37–7.39 (3H, m, 3′,4′,5′-H), 7.47 (1H, d, *J* = 2.4 Hz, 5-H), 7.52–7.54 (2H, m, 2′,6′-H), 7.59 (1H, d, *J* = 2.4 Hz, 5-H); ^13^C-NMR (125 MHz, DMSO-*d*_6_) 83.6, 97.0, 105.9, 112.0, 114.8, 122.4, 128.4, 128.9, 130.7, 131.6, 137.8, 149.1, 169.8; HRMS (ES): MH^+^, found 314.0050. C_15_H_11_N_2_O^79^Br^+^ requires 314.0055.

*2-Amino-5-bromo-3-(4-fluorophenylethynyl)benzamide* (**2b**). Solid (0.81 g, 84%), *R*_f_ (7:3 hexane–EtOAc) 0.61, m.p. 187–188 °C; IR (ATR): 533, 835, 1155, 1232, 1451, 1506, 1599, 1648, 1664, 3197, 3299, 3347, 3429 cm^−1^; ^1^H-NMR (500 MHz, DMSO-*d*_6_) 5.68 (2H, br s, NH_2_), 6.44 (2H, s, CONH_2_), 7.06–7.09 (2H, t, *J* = 8.7 Hz, 3′,5′-H), 7.47 (1H, d, *J* = 2.3 Hz, 5-H), 7.51 (2H, t, *J* = 8.7 Hz, 2′,6′-H), 7.57 (1H, d, *J* = 2.3 Hz, 5-H); ^13^C-NMR (125 MHz, DMSO-*d*_6_) 83.3, 95.8, 106.0, 111.8, 115.8 (d, ^2^*J*_CF_ = 22.9 Hz), 118.5 (d, ^4^*J*_CF_ = 3.7 Hz), 130.8, 133.5 (d, ^2^*J*_CF_ = 8.4 Hz), 137.8, 149.1, 162.8 (d, ^1^*J*_CF_ = 251.1 Hz), 169.8; HRMS (ES): MH^+^, found 331.9843. C_15_H_10_N_2_OF^79^Br^+^ requires 331.9882.

*2-Amino-5-bromo-3-(3-chlorophenylethynyl)benzamide* (**2c**). Solid (0.89 g, 87%), *R*_f_ (7:3 hexane–EtOAc) 0.63, m.p. 206–207 °C; IR (ATR): 554, 782, 876, 1092, 1243, 1402, 1558, 1601, 1648, 3298, 3347, 3428, 3452 cm^−1^; ^1^H-NMR (500 MHz, DMSO-*d*_6_) 6.92 (2H, s, -CONH_2_), 7.27 (1H, br s, NH), 7.31 (1H, d, *J* = 7.5 Hz, 4′-H), 7.32 (1H, dd, *J* = 1.5 and 7.5 Hz, 5′-H), 7.43 (1H, d, *J* = 2.5 Hz, 2′-H), 7.44 (1H, dd, *J* = 1.5 and 7.5 Hz, 6′-H), 7.64 (1H, d, *J* = 2.5 Hz, 6-H), 7.65 (1H, d, *J* = 2.5 Hz, 6-H), 7.88 (1H, br s, NH); ^13^C-NMR (125 MHz, DMSO-*d*_6_) 86.2, 94.6, 104.6, 109.8, 115.9, 124.5, 129.0, 130.3, 130.7, 131.2, 132.4, 133.4, 137.1, 149.8, 169.7; HRMS (ES): MH^+^, found 347.9652. C_15_H_10_N_2_O^35^Cl^79^Br^+^ requires 347.9665.

### 3.3. Typical Procedure for the Cyclocondensation of ***2a***–***c*** with Benzaldehyde Derivatives

A mixture of **2** (0.25 g, 7.93 mmol), benzaldehyde (1.68 g, 15.86 mmol) and boric acid (0.01 g, 1.58 mmol) was finely grounded and transferred into a round bottomed flask and then heated at 120 °C for 5 min. The resultant precipitate was washed thoroughly with cold water and recrystallized from ethanol to afford **3** as a solid. The following products were prepared in this fashion.

*6-Bromo-2-phenyl-8-(phenylethynyl)-2,3-dihydroquinazolin-4(1H)-one* (**3a**). Solid (1.10 g, 86%), m.p. 226–227 °C (EtOH); IR (ATR) 491, 697, 785, 833, 1238, 1485, 1508, 1592, 1683, 3175, 3382 cm^−1^; ^1^H-NMR (500 MHz, DMSO-*d*_6_) 5.84 (1H, t, *J* = 3.0 Hz, 2-H), 7.36 (1H, d, *J* = 2.0 Hz, 1-NH), 7.42–7.46 (8H, m, ArH), 7.63–7.65 (2H, m, ArH), 7.66 (1H, d, *J* = 2.5 Hz, 7-H), 7.68 (1H, d, *J* = 2.5 Hz, 5-H), 8.84 (1H, d, *J* = 2.5 Hz, 2-NH); ^13^C-NMR (125 MHz, DMSO-*d*_6_) 65.0, 83.8, 96.7, 107.9, 110.2, 117.0, 122.4, 128.4, 129.0, 129.1, 129.6, 130.7, 132.2, 133.3, 138.4, 141.9, 146.6, 161.6; HRMS (ES): MH^+^, found 403.0457. C_22_H_16_N_2_O^79^Br^+^ requires 403.0446.

*6-Bromo-2-(4-fluorophenyl)-8-(phenylethynyl)-2,3-dihydroquinazolin-4(1H)-one* (**3b**). Solid (1.18 g, 88%), m.p. 235–237 °C (EtOH); IR (ATR): 557, 685, 753, 1230, 1488, 1589, 1681, 3372, 3477 cm^−1^; ^1^H-NMR (500 MHz, DMSO-*d*_6_) 5.85 (1H, t, *J* = 3.0 Hz, 2-H), 7.21 (2H, t, *J* = 8.7 Hz, 3′,5′-H), 7.34 (1H, d, *J* = 2.0 Hz, 1-NH), 7.43–7.47 (5H, m, ArH), 7.64 (2H, t, *J* = 8.7 Hz, 2′,6′-H), 7.66 (1H, d, *J* = 2.5 Hz, 5-H), 7.69 (1H, d, *J* = 2.5 Hz, 5-H), 8.84 (1H, d, *J =* 2.5 Hz, 3-NH); ^13^C-NMR (125 MHz, DMSO-*d*_6_) 65.0, 83.8, 96.7, 107.8, 110.2, 115.4 (d, ^2^*J*_CF_ = 20.6 Hz), 117.1, 122.4, 128.5 (d, ^3^*J*_CF_ = 8.6 Hz), 129.1, 129.6, 130.7, 132.2, 138.8 (d, ^4^*J*_CF_ = 3.0 Hz), 139.1, 146.7, 161.6, 162.4 (d, ^1^*J*_CF_ = 243.8 Hz); HRMS (ES): MH^+^, found 421.0342. C_22_H_14_N_2_OF^79^Br^+^ requires 421.0352.

*6-Bromo-2-(4-chlorophenyl)-8-(phenylethynyl)-2,3-dihydroquinazolin-4(1H)-one* (**3c**). Solid (1.20 g, 86%), m.p. 231–232 °C (EtOH); IR (ATR): 475, 532, 685, 752, 1279, 1382, 1486, 1587, 1682, 3176, 3380 cm^−1^; ^13^C-NMR (500 MHz, DMSO-*d*_6_) 5.84 (1H, t, *J* = 3.0 Hz, 2-H), 7.28 (1H, d, *J* = 2.0 Hz, 1-NH), 7.37 (2H, d, *J* = 7.5 Hz, 3′,5′-H), 7.38 (2H, d, *J* = 7.5 Hz, 2′,6′-H), 7.41–7.44 (3H, m, ArH), 7.64–7.65 (2H, m, ArH), 7.66 (1H, d, *J* = 2.5 Hz, 7-H), 7.69 (1H, d, *J* = 2.5 Hz, 5-H), 8.84 (1H, d, *J* = 3.0 Hz, 3-NH); ^13^C-NMR (125 MHz, DMSO-*d*_6_) 65.5, 83.9, 96.7, 107.6, 110.1, 117.1, 122.4, 126.3, 128.6, 128.9, 129.1, 129.5, 130.7, 132.1, 138.3, 143.1, 146.7, 161.7; HRMS (ES): MH^+^, found 437.0053. C_22_H_15_N_2_O^35^Cl^79^Br^+^ requires 437.0053.

*6-Bromo-2-(4-methoxyphenyl)-8-(phenylethynyl)-2,3-dihydroquinazolin-4(1H)-one* (**3d**). Solid (1.31 g, 93%), m.p. 254–256 °C (EtOH); IR (ATR): 534, 686, 751, 1040, 1241, 1378, 1516, 1586, 1680, 3176, 3382 cm^−1^; ^1^H-NMR (500 MHz, DMSO-*d*_6_) 3.71 (3H, s, OCH_3_), 5.79 (1H, t, *J* = 3.5 Hz, 2-H), 6.91 (2H, d, *J* = 8.5 Hz, 3′,5′-H), 7.24 (1H, d, *J* = 2.0 Hz, 7-H), 7.24 (2H, d, *J* = 8.5 Hz, 2′,6′-H), 7.42–7.45 (3H, m, ArH), 7.63–7.65 (3H, m, 1-NH and ArH), 7.67 (1H, d, *J* = 2.0 Hz, 5-H), 8.76 (1H, d, *J* = 3.5 Hz, 3-NH); ^13^C-NMR (125 MHz, DMSO-*d*_6_) 55.6, 65.2, 83.9, 96.6, 107.6, 110.1, 114.3, 117.2, 122.4, 127.6, 129.1, 129.5, 130.6, 132.1, 134.9, 138.3, 146.8, 159.6, 161.8; HRMS (ES): MH^+^, found 433.0553. C_23_H_18_^79^BrN_2_O_2_^+^ requires 433.0553.

*6-Bromo-8-((4-fluorophenyl)ethynyl)-2-phenyl-2,3-dihydroquinazolin-4(1H)-one* (**3e**). Solid (1.15 g, 91%), m.p. 313 °C (EtOH); IR (ATR): 538, 686, 751, 1017, 1090, 1377, 1483, 1589, 1679, 3182, 3379 cm^−1^; ^1^H-NMR (500 MHz, DMSO-*d*_6_) 5.84 (1H, t, *J* = 3.5 Hz, 2-H), 7.30 (2H, t, *J* = Hz, 3′,5′-H), 7.31 (1H, d, *J* = 2.0 Hz, 1-NH), 7.35–7.38 (3H, m, ArH), 7.41 (2H, d, *J* = 7.0 Hz, ArH), 7.64 (1H, d, *J* = 2.5 Hz, 7-H), 7.68 (1H, d, *J* = 2.5 Hz, 5-H), 7.71 (2H, t, *J* = 8.5 Hz, 2′,6′-H), 8.84 (1H, d, *J* = 3.5 Hz, 3-NH); ^13^C-NMR (125 MHz, DMSO-*d*_6_) 65.5, 83.6, 95.6, 107.6, 109.9, 115.3 (d, ^2^*J*_CF_ = 21.7 Hz), 117.1, 118.9 (d, ^4^*J*_CF_ = 3.3 Hz), 126.3, 128.6, 128.9, 130.7, 134.5 (d, ^3^*J*_CF_ = 8.5 Hz), 138.3, 143.0, 146.8, 161.7, 162.7 (d, ^1^*J*_CF_ = 246.0 Hz); HRMS (ES): MH^+^, found 421.0349. C_22_H_15_N_2_OF^79^Br^+^ requires 421.0352.

*6-Bromo-2-(4-fluorophenyl)-8-((4-fluorophenyl)ethynyl)-2,3-dihydroquinazolin-4(1H)-one* (**3f**). Solid (1.22 g, 92%), m.p. 286–287 °C (EtOH); IR (ATR): 491, 765, 783, 832, 1158, 1235, 1490, 1509, 1590, 1682, 3179, 3374 cm^−1^; ^1^H-NMR (500 MHz, DMSO-*d*_6_) 5.85 (1H, t, *J* = 3.5 Hz, 2-H), 7.31 (2H, t, *J* = 8.5 Hz, 3′,5′-H), 7.39 (1H, d, *J* = 3.0 Hz, 1-NH), 7.44 (2H, t, *J* = 8.5 Hz, 2′,6′-H), 7.45 (2H, t, *J* = 8.5 Hz, 3″,5″-H), 7.66 (1H, d, *J* = 2.0 Hz, 7-H), 7.69 (1H, d, *J* = 2.0 Hz, 5-H), 7.71 (2H, t, *J =* 8.5 Hz, 2″,6″-H), 8.84 (1H, d, *J* = 3.5 Hz, 3-NH); ^13^C-NMR (125 MHz, DMSO-*d*_6_) 65.0, 83,6, 95.6, 107.8, 110.1, 115.7 (d, ^2^*J*_CF_ = 21.0 Hz), 116.3 (d, ^2^*J*_CF_ = 22.7 Hz), 117.1, 118.9 (d, ^4^*J*_CF_ = 3.8 Hz), 128.5 (d, ^3^*J*_CF_ = 8.6 Hz), 130.7, 134.5 (d, ^3^*J*_CF_ = 8.5 Hz), 138.4, 139.1 (d, ^4^*J*_CF_ = 2.9 Hz), 146.7, 161.6, 162.4 (d, ^1^*J*_CF_ = 244.5 Hz), 162.7 (d, ^1^*J*_CF_ = 246.5 Hz); HRMS (ES): MH^+^, found 439.0241. C_22_H_13_N_2_OF_2_^79^Br^+^ requires 439.0258.

*6-Bromo-2-(4-chlorophenyl)-8-((4-fluorophenyl)ethynyl)-2,3-dihydroquinazolin-4(1H)-one* (**3g**). Solid (1.28 g, 93%), m.p. 275–276 °C (EtOH); IR (ATR): 496, 329, 1091, 1228, 1379, 1483, 1507, 1588, 1681, 3172, 3379 cm^−1^; ^1^H-NMR (500 MHz, DMSO-*d*_6_) 5.85 (1H, t, *J* = 2.5 Hz, 2-H), 7.31 (2H, t, *J* = 8.5 Hz, 3′,5′-H), 7.39 (1H, d, *J* = 2.0 Hz, 1-NH), 7.43 (2H, d, *J* = 8.5 Hz, 3″,5″-H), 7.45 (2H, d, *J* = 8.5 Hz, 2″,6″-H), 7.66 (1H, d, *J* = 2.5 Hz, 7-H), 7.69 (1H, d, *J* = 2.5 Hz, 5-H) 7.71 (2H, d, *J* = 8.5 Hz, 2′,6′-H), 8.84 (1H, d, *J* = 2.5 Hz, 3-NH); ^13^C NMR (125 MHz, DMSO-*d*_6_) 65.0, 83,6. 95.7, 107.8, 110.1, 116.3 (d, ^2^*J*_CF_ = 21.9 Hz), 117.1, 118.9 (d, ^4^*J*_CF_ = 3.5 Hz), 128.4, 129.0, 130.7, 133.3, 134.5 (d, ^3^*J*_CF_ = 8.5 Hz), 138.4, 141.9, 146.6, 161.6, 162.7 (d, ^1^*J*_CF_ = 248.6 Hz); HRMS (ES): MH^+^, found 455.9967. C_22_H_14_N_2_OF^35^Cl^79^Br^+^ requires 455.9962.

*6-Bromo-8-((4-fluorophenyl)ethynyl)-2-(4-methoxyphenyl)-2,3-dihydroquinazolin-4(1H)-one* (**3h**). Solid (1.24 g, 92%), m.p. 215–216 °C (EtOH); ν_max_ (ATR) 443, 648, 781, 883, 1041, 1178, 1489, 1586, 1684, 3179, 3390 cm^−1^; ^1^H-NMR (500 MHz, DMSO-*d*_6_) 3.69 (3H, s, OCH_3_), 5.77 (1H, t, *J* = 3.0 Hz, 2-H), 6.89 (2H, d, *J* = 8.5 Hz, 3″,5″-H), 7.27 (1H, d, *J* = 2.2 Hz, 1-NH), 7.28 (2H, t, *J* = 8.7 Hz, 3′,5′-H), 7.61 (2H, d, *J* = 8.5 Hz, 2″,6″-H), 7.62 (1H, d, *J* = 3.0 Hz, 8-H), 7.66 (1H, d, *J* = 3.0 Hz, 5-H), 7.69 (2H, t, *J* = 8.7 Hz, 2′,6′-H), 8.75 (1H, d, *J* = 3.0 Hz, 3-NH); ^13^C-NMR (125 MHz, DMSO-*d*_6_) 55.6, 65.1, 83.7, 95.5, 107.6, 109.9, 114.2, 116.3 (d, ^2^*J*_CF_ = 21.8 Hz), 117.2, 118.9 (d, ^4^*J*_CF_ = 3.6 Hz), 127.6, 130.7, 134. 4, 134.4 (d, ^3^*J*_CF_ = 8.5 Hz), 134.9, 138.2, 146.8, 159.6, 161.7 (d, ^1^*J*_CF_ = 246.6 Hz); HRMS (ES): MH^+^, found 451.0457. C_23_H_17_N_2_O_2_F^79^Br^+^ requires 451.0457.

*6-Bromo-8-((3-chlorophenyl)ethynyl)-2-phenyl-2,3-dihydroquinazolin-4(1H)-one* (**3i**). Solid (1.00 g, 80%); m.p. 218–219 °C (EtOH); IR (ATR): 678, 780, 873, 1289, 1487, 1592, 1682, 3172, 3379 cm^−1^; ^1^H-NMR (500 MHz, DMSO-*d*_6_) 5.84 (1H, t, *J* = 2.5 Hz, 2-H), 7.20 (2H, t, *J* = 8.5 Hz, ArH), 7.42–7.50 (6H, m, ArH), 7.58 (1H, dd, *J* = 2.5 and 7.5 Hz, 5′-H), 7.67 (1H, d, *J* = 2.0 Hz, 7-H), 7.68 (1H, d, *J* = 2.0 Hz, 5-H), 7.73 (1H, s, 1-NH), 8.84 (1H, d, *J* = 2.5 Hz, 3-NH); ^13^C-NMR (125 MHz, DMSO-*d*_6_) 65.5, 85.2, 95.0, 107.6, 109.5, 117.1, 124.5, 126.3, 128.7, 129.0, 129.5, 130.7, 130.9, 131.0, 131.5, 133.6, 138.5, 143.0, 146.9, 161.6; HRMS (ES): MH^+^, found 437.0049. C_22_H_14_N_2_O^35^Cl^79^Br^+^ requires 437.0057.

*6-Bromo-8-((3-chlorophenyl)ethynyl)-2-(4-fluorophenyl)-2,3-dihydroquinazolin-4(1H)-one* (**3j**). Solid (1.23 g, 95%), m.p. 211–212 °C (EtOH); IR (ATR): 529, 752, 1229, 1381, 1486, 1589, 1681, 3179, 3375 cm^−1^; ^1^H-NMR (500 MHz, DMSO-*d*_6_) 5.83 (1H, t, *J* = 2.5 Hz, 2-H), 7.20 (2H, t, *J* = 8.7 Hz, 3″,5″-H), (1H, d, *J* = 2.5 Hz, -NH), 7.41–7.44 (3H, m, ArH), 7.63 (2H, t, *J =* 8.7 Hz, 2″,6″-H), 7.66–7.68 (2H, m, ArH), 7.90 (1H, d, *J* = 2.0 Hz, 5-H), 8.81 (1H, d, *J* = 2.5 Hz, 3-NH); ^13^C-NMR (125 MHz, DMSO-*d*_6_) 65.0, 83.8, 96.7, 107.8, 110.2, 115.6 (d, ^2^*J*_CF_ = 21.9 Hz), 117.0, 122.4, 128.4, 128.6 (d, ^3^*J*_CF_ = 8.5 Hz), 129.1, 129.4, 129.6, 130.7, 132.1, 138.4, 139.1 (d, ^4^*J*_CF_ = 2.9 Hz), 146.6, 161.6, 162.3 (d, ^1^*J*_CF_ = 242.8 Hz); HRMS (ES): MH^+^, found 454.9970. C_22_H_13_N_2_OF^35^Cl^79^Br^+^ requires 454.9962.

*6-Bromo-2-(4-chlorophenyl)-8-((3-chlorophenyl)ethynyl)-2,3-dihydroquinazolin-4(1H)-one* (**3k**). Solid (1.30 g, 96%), m.p. 214–215 °C (EtOH); IR (ATR): 540, 676, 777, 1089, 1379, 1484, 1589, 1684, 3175, 3373 cm^−1^; ^1^H-NMR (500 MHz, DMSO-*d*_6_) 5.84 (1H, t, *J* = 2.5 Hz, 2-H), 7.40–7.50 (7H, m, ArH), 7.58 (1H, dd, *J* = 2.5 and 7.5 Hz, 5′-H), 7.67 (1H, d, *J* = 2.5 Hz, 7-H), 7.68 (1H, d, *J* = 2.5 Hz, 5-H), 7.77 (1H, s, 1-NH), 8.86 (1H, d, *J* = 2.5 Hz, 3-NH); ^13^C-NMR (125 MHz, DMSO-*d*_6_) 64.9, 85.1, 95.1, 107.8, 109.6, 117.1, 124.4, 128.3, 129.0, 129.6, 130.7, 131.0, 131.1, 131.5, 133.3, 133.6, 138.6, 141.8, 146.7, 161.5; HRMS (ES): MH^+^, found 470.9651. C_22_H_14_N_2_O^35^Cl_2_^79^Br^+^ requires 470.9667.

*6-Bromo-8-((3-chlorophenyl)ethynyl)-2-(4-methoxyphenyl)-2,3-dihydroquinazolin-4(1H)-one* (**3l**). Solid (1.30 g, 97%), m.p. 200–201 °C (EtOH); IR (ATR): 499, 826, 1037, 1230, 1253, 1384, 1484, 1508, 1588, 1685, 3177, 3379 cm^−1^; ^1^H-NMR (500 MHz, DMSO-*d*_6_) 3.70 (3H, s, OCH_3_), 5.78 (1H, t, *J* = 3.0 Hz, 2-H), 6.91 (2H, d, *J* = 8.5 Hz, 3″,5″-H), 7.31 (2H, d, *J* = 8.5 Hz, 2″,6″-H), 7.35 (1H, d, *J* = 2.0 Hz, 1-NH), 7.45 (1H, d, *J* = 7.5 Hz, 4′-H), 7.48 (1H, d, *J* = 6.5 Hz, 2′-H), 7.81 (1H, dd, *J* = 2.0 and 7.5 Hz, 5′-H), 7.58 (1H, d, *J* = 7.5 Hz, 6′-H), 7.65 (1H, d, *J* = 2.5 Hz, 7-H), 7.78 (1H, d, *J* = 2.5 Hz, 5-H), 8.79 (1H, d, *J* = 3.0 Hz, 3-NH); ^13^C-NMR (125 MHz, DMSO-*d*_6_) 55.6, 65.2, 85.3, 95.0, 107.5, 109.4, 114.3, 117.2, 124.5, 127.6, 129.5, 130.7, 130.9, 131.0, 131.5, 133.6, 134.9, 138.5, 146.9, 159.6, 161.6; HRMS (ES): MH^+^, found 467.0160. C_23_H_16_N_2_O_2_^35^Cl^79^Br^+^ requires 467.0162.

### 3.4. Typical Procedure for the PdCl_2_-Mediated Heteroannulation of ***3a***–***l*** to Afford ***4a***–***l***

A stirred mixture of **3a** (0.50 g, 1.24 mmol) and PdCl_2_ (0.03 g, 0.25 mmol) in dioxane (20 mL) was heated at 90 °C for 2 h. The mixture was allowed to cool to room temperature and then quenched with an ice-cold water. The precipitate was filtered and dissolved in chloroform. The organic layer was washed with water, dried over Na_2_SO_4_, filtered and evaporated under reduced pressure. The residue was purified by column chromatography on silica gel to afford **4a**. The following products were prepared in this fashion:

*8-Bromo-3,5-diphenyl-2,3-dihydro-1H-pyrrolo[3,2,1-ij]quinazolin-1-one* (**4a**). Solid (0.29 g, 58%), *R*_f_ (8:2 hexane–EtOAc) 0.21, m.p. 186–187 °C; IR (ATR): 527, 694, 755.0, 1276, 1456, 1647, 3074, 3185 cm^−1^; ^1^H-NMR (500 MHz, DMSO-*d*_6_) 6.53 (2H, d, *J* = 7.0 Hz, ArH), 6.77 (1H, s, 6-H), 7.05 (1H, d, *J* = 3.5 Hz, 3-H), 7.06–7.20 (3H, m, ArH), 7.34–7.42 (3H, m, ArH), 7.56 (2H, d, *J* = 7.0 Hz, ArH), 7.60 (1H, d, *J* = 1.5 Hz, 7-H), 8.00 (1H, d, *J* = 1.5 Hz, 9-H), 9.16 (1H, d, *J* = 3.0 Hz, NH); ^13^C-NMR (125 MHz, DMSO-*d*_6_) 70.2, 104.1, 113.7, 115.6, 121.5, 125.2, 127.2, 128.4, 128.5, 129.1, 129.2, 129.3, 129.4, 131.1, 137.3, 141.3, 142.6, 160.3; HRMS (ES): MH^+^, found 403.0457. C_22_H_16_^79^BrN_2_O^+^ requires 403.0446.

*8-Bromo-3-(4-fluorophenyl)-5-phenyl-2,3-dihydro-1H-pyrrolo[3,2,1-ij]quinazolin-1-one* (**4b**). Solid (0.25 g, 53%), *R*_f_ (8:2 hexane–EtOAc) 0.21, m.p. 185–186 °C; IR (ATR): 524, 701, 765, 842, 1224, 1474, 1675, 3076, 3189 cm^−1^; ^1^H-NMR (300 MHz, DMSO-*d*_6_) 6.56 (2H, d, *J =* 8.5 Hz, 3′,5′-H), 6.92 (1H, s, 6-H), 7.08 (1H, d, *J* = 3.5 Hz, 3-H), 7.35–7.43 (3H, m, ArH), 7.56 (2H, dd, *J* = 1.8 and 8.1 Hz, ArH), 7.61 (1H, d, *J* = 1.5 Hz, 7-H), 8.01 (1H, d, *J* = 1.5 Hz, 9-H), 9.16 (1H, d, *J* = 3.0 Hz, NH); ^13^C-NMR (75 MHz, DMSO-*d*_6_) 69.6, 104.2, 113.7, 115.3, 116.0 (d, ^2^*J*_CF_ = 21.9 Hz), 121.6, 127.4, 127.5 (d, ^3^*J*_CF_ = 8.5 Hz), 128.4, 128.5, 129.3, 129.5, 130.9, 137.1, 137.6 (d, ^4^*J*_CF_ = 2.9 Hz), 142.5, 160.3, 162.3 (d, ^1^*J*_CF_ = 246.3 Hz); HRMS (ES): MH^+^, found 421.0342. C_22_H_15_N_2_OF^79^Br^+^ requires 421.0352.

*8-Bromo-3-(4-chlorophenyl)-5-phenyl-2,3-dihydro-1H-pyrrolo[3,2,1-ij]quinazolin-1-one* (**4c**). Solid (0.31 g, 62%), *R*_f_ (8:2 hexane-EtOAc) 0.23, m.p. 208–209 °C; IR (ATR): 455, 517, 697, 757, 851, 1110, 1323, 1469, 1676, 3072, 3182 cm^−1^; ^1^H-NMR (500 MHz, DMSO-*d*_6_) 6.54 (2H, d, *J* = 8.5 Hz, 3′,5′-H), 6.78 (1H, s, 6-H), 7.08 (1H, d, *J* = 3.5 Hz, 3-H), 7.16 (2H, d, *J* = 8.5 Hz, 2′,6′-H), 7.37–7.43 (3H, m, ArH), 7.57 (2H, d, *J* = 7.0 Hz, ArH), 7.61 (1H, d, *J* = 1.5 Hz, 7-H), 8.02 (1H, d, *J* = 1.5 Hz, 9-H), 9.16 (1H, d, *J* = 3.0 Hz, NH); ^13^C-NMR (125 MHz, DMSO-*d*_6_) 69.6, 104.2, 113.8, 115.4, 121.6, 127.2, 127.3, 128.4, 128.5, 129.1, 129.3, 129.5, 130.9, 133.7, 137.1, 140.2, 142.5, 160.2; HRMS (ES): MH^+^, found 437.0053. C_22_H_15_N_2_O^35^Cl^79^Br^+^ requires 437.0056.

*8-Bromo-3-(4-methoxyphenyl)-5-phenyl-2,3-dihydro-1H-pyrrolo[3,2,1-ij]quinazolin-1-one* (**4d**). Solid (0.30 g, 60%), *R*_f_ (8:2 hexane–EtOAc) 0.19, m.p. 174–175 °C; IR (ATR): 526, 573, 687, 759, 1032, 1174, 1246, 1465, 1512, 1614, 1669, 3074, 3182 cm^−1^; ^1^H-NMR (300 MHz, DMSO-*d*_6_) 3.57 (3H, s, OCH_3_), 6.45 (2H, d, *J =* 8.7 Hz, 3′,5′-H), 6.62 (2H, d, *J =* 8.7 Hz, 2′,6′-H), 6.70 (1H, s, 6-H), 7.00 (1H, d, *J* = 3.5 Hz, 3-H), 7.33–7.45 (3H, m, ArH), 7.58 (2H, d, *J* = 7.0 Hz, ArH), 7.99 (1H, d, *J* = 1.5 Hz, 9-H), 9.11 (1H, d, *J* = 3.0 Hz, NH); ^13^C-NMR (125 MHz, DMSO-*d*_6_) 5.4, 69.9, 104.1, 113.6, 114.3, 121.4, 126.5, 127.2, 127.8, 128.4, 128.5, 129.2, 129.5, 131.2, 133.6, 137.2, 142.6, 159.7, 160.4; HRMS (ES): MH^+^, found 433.0553. C_23_H_18_N_2_O_2_^79^Br^+^ requires 433.0552.

*8-Bromo-5-(4-fluorophenyl)-3-phenyl-2,3-dihydro-1H-pyrrolo[3,2,1-ij]quinazolin-1-one* (**4e**). Solid (0.27 g, 57%), *R*_f_ (8:2 hexane–EtOAc) 0.26, m.p. 220–222 °C; IR (ATR): 518, 563, 694, 775, 845, 1226, 1319, 1492, 1607, 1672, 3065, 3185 cm^−1^; ^1^H-NMR (500 MHz, DMSO-*d*_6_) 6.55 (2H, d, *J* = 7.0 Hz, ArH), 6.74 (1H, s, 6-H), 7.03 (1H, d, *J* = 3.0 Hz, 3-H), 7.07–7.14 (3H, m, ArH), 7.22 (2H, t, *J* = 8.7 Hz, 3′,5′-H),7.60 (2H, t, *J* = 8.7 Hz, 2′,6′-H), 7.61 (1H, d, *J* = 1.5 Hz, 6-H), 8.00 (1H, d, *J* = 1.5 Hz, 9-H), 9.16 (1H, d, *J* = 3.0 Hz, NH); ^13^C-NMR (125 MHz, DMSO-*d*_6_) 64.9, 85,2, 95.0, 107.7, 109.6, 115.7 (d, ^2^*J*_CF_ = 21.8 Hz), 117.1, 124.4, 128.5 (d, ^3^*J*_CF_ = 8.5 Hz), 129.5, 130.7, 130.9, 131.1, 131.5, 133.6, 138.6, 139.1 (d, ^4^*J*_CF_ = 2.9 Hz), 146.8, 161.5, 162.4 (d, ^1^*J*_CF_ = 242.8 Hz); HRMS (ES): MH^+^, found 421.0341. C_22_H_14_N_2_OF^79^Br^+^ requires 421.0352.

*8-Bromo-3,5-bis(4-fluorophenyl)-2,3-dihydro-1H-pyrrolo[3,2,1-ij]quinazolin-1-one* (**4f**). Solid (0.28 g, 56%), *R*_f_ (8:2 hexane–EtOAc) 0.26, m.p. 239–240 °C; IR (ATR): 510, 566, 772, 835, 1157, 1225, 1320, 1494, 1509, 1620, 1671, 3073, 3187 cm^−1^; ^1^H-NMR (500 MHz, DMSO-*d*_6_) 6.54 (1H, s, 6-H), 6.59 (2H, d, *J =* 8.5 Hz, 3′,5′-H), 6.74 (2H, d, *J =* 8.5 Hz, 3″,5″-H), 6.85 (1H, d, *J* = 3.5 Hz, 3-H), 7.08 (2H, d, *J =* 8.5 Hz, 2′,6′-H), 7.29 (2H, d, *J =* 8.5 Hz, 2″,6″-H), 7.86 (1H, d, *J =* 1.5 Hz, 7-H), 7.80 (1H, d, *J* = 1.5 Hz, 9-H), 7.95 (1H, d, *J* = 2.0 Hz, 3-NH); ^13^C-NMR (125 MHz, DMSO-*d*_6_) 70.3, 104.2, 114.4, 115.9 (d, ^2^*J*_CF_ = 21.9 Hz), 116.1 (d, ^2^*J*_CF_ = 20.9 Hz), 122.9, 127.0 (d, ^3^*J*_CF_ = 8.5 Hz), 127.1 (d, ^4^*J*_CF_ = 3.8 Hz), 127.8 (2C), 128.1, 130.3 (d, ^3^*J*_CF_ = 8.5 Hz), 135.9 (d, ^4^*J*_CF_ = 3.8 Hz), 136.8, 140.8, 162.0, 162.8 (d, ^1^*J*_CF_ = 248.4 Hz), 163.0 (d, ^1^*J*_CF_ = 249.3 Hz); HRMS (ES): MH^+^, found 439.0241. C_22_H_13_N_2_OF_2_^79^Br^+^ requires 439.0258.

*8-Bromo-3-(4-chlorophenyl)-5-(4-fluorophenyl)-2,3-dihydro-1H-pyrrolo[3,2,1-ij]quinazolin-1-one* (**4g**). Solid (0.32 g, 58%), *R*_f_ (8:2 hexane–EtOAc) 0.27, m.p. 216–217 °C; IR (ATR): 510.0, 563, 812, 824, 1088, 1160, 1225, 1491, 1620, 1672, 3074, 3185 cm^−1^. ^1^H-NMR (500 MHz, DMSO-*d*_6_) 6.56 (2H, d, *J =* 7.5 Hz, 3″,5″-H), 7.76 (1H, s, 6-H), 7.05 (1H, d, *J =* 2.5 Hz, 3-H), 7.18 (2H, d, *J =* 7.5 Hz, 2″,6"-H), 7.24 (2H, t, *J* = 8.5 Hz, 3′,5′-H), 7.61 (2H, t, *J* = 8.5 Hz, 2′,6′-H), 7.62 (1H, d, *J* = 1.5 Hz, 7-H), 8.01 (1H, d, *J* = 1.5 Hz, 9-H), 9.17 (1H, d, *J =* 2.5 Hz, 3-NH); ^13^C-NMR (125 MHz, DMSO-*d*_6_) 70.3, 104.1, 113.7, 115.5, (116.4 (d, ^2^*J*_CF_ = 21.9Hz), 121.5, 127.2, 127.6 (d, ^3^*J*_CF_ = 3.8 Hz), 128.4, 129.1 (2C), 129.2, 130.8 (d, ^3^*J*_CF_ = 8.5 Hz), 137.1, 141.2, 141.5, 160.2, 162.7 (d, ^1^*J*_CF_ = 245.5 Hz); HRMS (ES): MH^+^, found 455.9970. C_22_H_13_N_2_OF^35^Cl^79^Br^+^ requires 454.9962.

*8-Bromo-5-(4-fluorophenyl)-3-(4-methoxyphenyl)-2,3-dihydro-1H-pyrrolo[3,2,1-ij]quinazolin-1-one* (**4h**). Solid (0.30 g, 60%), *R*_f_ (8:2 hexane–EtOAc) 0.24, m.p. 200–201 °C; IR (ATR): 511, 590, 837, 1174, 1246, 1466, 1493, 1620, 1670, 3071, 3184 cm^−1^; ^1^H-NMR (500 MHz, DMSO-*d*_6_) 3.58 (3H, s, OCH_3_), 6.46 (2H, d, *J* = 8.5 Hz, 3",5"-H), 6.64 (2H, d, *J* = 8.0 Hz, 2″,6″-H), 6.74 (1H, s, 6-H), 6.97 (1H, d, *J* = 1.5 Hz, 3-H), 7.24 (2H, t, *J* = 8.7 Hz, 3′,5′-H), 7.59 (1H, d, *J* = 1.5 Hz, 9-H), 7.61 (2H, t, *J* = 8.7 Hz, 2′,6′-H), 7.98 (1H, d, *J* = 1.5 Hz, 5-H), 9.10 (1H, d, *J* = 3.5 Hz, 3-NH); ^13^C-NMR (125 MHz, DMSO-*d*_6_) 55.5, 69.9, 104.1, 113.6, 114.3, 115.5, 116.4 (d, ^2^*J*_CF_ = 21.8 Hz), 121.4, 126.6, 127.2, 127.7 (d, ^4^*J*_CF_ = 2.9 Hz), 128.3, 130.8 (d, ^3^*J*_CF_ = 8.8 Hz), 133.5, 137.1, 141.5, 159.7, 160.2, 162.6 (d, *J*_CF_ = 250.5 Hz); HRMS (ES): MH^+^, found 451.0457. C_23_H_17_^79^BrFN_2_O_2_^+^ requires 451.0457.

*8-Bromo-5-(3-chlorophenyl)-3-phenyl-2,3-dihydro-1H-pyrrolo[3,2,1-ij]quinazolin-1-one* (**4i**). Solid (0.34 g, 68%), *R*_f_ (8:2 hexane–EtOAc) 0.24, m.p. 234–235 °C; IR (ATR) 517, 534, 692, 773, 1269, 1318, 1456, 1621, 1677, 3062, 3176 cm^−1^; ^1^H-NMR (500 MHz, DMSO-*d*_6_) 6.56 (2H, d, *J* = 7.0 Hz, ArH), 6.84 (1H, s, 6-H), 7.08 (1H, d, *J* = 1.5 Hz, 3-H), 7.10–7.16 (4H, m, ArH), 7.41–7.43 (2H, m, 4′-H and 6′-H), 7.54 (1H, dd, *J* = 2.0 and 3.5 Hz, 5′-H), 7.63 (1H, d, *J* = 1.5 Hz, 7-H), 8.02 (1H, d, *J* = 1.5 Hz, 9-H), 9.15 (1H, d, *J* = 3.5 Hz, NH); ^13^C-NMR (125 MHz, DMSO-*d*_6_) 70.3, 104.9, 113.8, 115.6, 121.9, 125.3, 127.2, 127.4, 128.0, 128.2, 129.1, 129.2, 129.3, 131.2, 133.1, 134.0, 137.3, 140.8, 141.2, 160.0; m/z 437 (100, MH^+^); HRMS (ES): MH^+^, found 437.0049. C_22_H_15_N_2_O^35^Cl^79^Br^+^ requires 437.0056.

*8-Bromo-5-(3-chlorophenyl)-3-(4-fluorophenyl)-2,3-dihydro-1H-pyrrolo[3,2,1-ij]quinazolin-1-one* (**4j**). Solid (0.34 g, 62%), *R*_f_ (8:2 hexane-EtOAc) 0.27, m.p. 227–228 °C; IR (ATR): 529, 566, 692.1, 772, 788, 1148, 1173, 1232, 1319, 1461, 1508, 1599, 1671, 3073, 3187 cm^−1^; ^1^H-NMR (500 MHz, DMSO-*d*_6_) 6.60 (2H, t, *J* = 8.5 Hz, 3″,5″-H), 6.84 (1H, s, 6-H), 6.95 (2H, t, *J* = 8.5 Hz, 2″,6″-H), 7.11 (1H, d, *J* = 3.5 Hz, 3-H), 7.41–7.43 (2H, m, 4′-H and 6′-H), 7.34 (1H, dd, *J* = 1.5 and 3.5 Hz, 5′-H), 7.62 (1H, d, *J* = 1.5 Hz, 7-H), 7.72 (1H, s, 2′-H), 8.02 (1H, d, *J* = 1.5 Hz, 9-H), 9.14 (1H, d, *J* = 3.0 Hz, NH); ^13^C-NMR (125 MHz, DMSO-*d*_6_) 69.7, 105.0, 113.8, 115.4, 116.0 (d, ^2^*J*_CF_ = 21.8 Hz), 121.9, 127.2, 127.5, 127.6 (d, ^3^*J*_CF_ = 85 Hz), 128.0, 128.2, 129.1, 131.2, 133.0, 134.0, 137.1, 137.5 (d, ^3^*J_CF_* = 2.9 Hz), 140.6, 159.9, 162.3 (d, ^1^*J*_CF_ = 243.6 Hz); HRMS (ES): MH^+^, found 454.9970. C_22_H_14_N_2_OF^35^Cl^79^Br ^+^ requires 454.9962.

*8-Bromo-5-(3-chlorophenyl)-3-(4-chlorophenyl)-2,3-dihydro-1H-pyrrolo[3,2,1-ij]quinazolin-1-one* (**4k**). Solid (0.38 g, 76%), *R*_f_ (8:2 hexane–EtOAc) 0.29, m.p. 238–239 °C; IR (ATR): 523, 773, 1089, 1318, 1464, 1599, 1677, 3069, 3178 cm^−1^; ^1^H-NMR (500 MHz, DMSO-*d*_6_) 6.57 (2H, d, *J* = 8.5 Hz, 3″,5″-H), 6.86 (1H, s, 6-H), 7.11 (1H, d, *J* = 3.5 Hz, 3-H), 7.20 (2H, d, *J* = 8.5 Hz, 2″,6″-H), 7.41–7.43 (2H, m, 4′-H and 6′-H), 7.54 (1H, dd, *J* = 1.5 and 3.5 Hz, 5′-H), 7.62 (1H, d, *J* = 1.5 Hz, 7-H), 7.65 (1H, s, 2′-H), 8.03 (1H, d, *J* = 1.5 Hz, 9-H), 9.16 (1H, d, *J* = 3.0 Hz, NH); ^13^C-NMR (125 MHz, DMSO-*d*_6_) 69.7, 105.1, 113.9, 115.4, 122.0, 127.2, 127.3, 127.6, 128.0, 128.2, 129.2, 129.3, 131.3, 133.0, 133.8, 134.0, 137.1, 140.1, 140.6, 159.9; HRMS (ES): MH^+^, found 470.9651. C_22_H_13_N_2_O^35^Cl_2_^79^Br^+^ requires 470.9667.

*8-Bromo-5-(3-chlorophenyl)-3-(4-methoxyphenyl)-2,3-dihydro-1H-pyrrolo[3,2,1-ij]quinazolin-1-one* (**4l**). Solid (0.36 g, 72%), *R*_f_ (8:2 hexane–EtOAc) 0.22, m.p. 177–178 °C; IR (ATR): 514, 572, 772, 1033, 1175, 1247, 1456, 1512, 1598, 1671, 3068, 3187 cm^−1^; ^1^H-NMR (500 MHz, DMSO-*d*_6_) 3.58 (3H, s, OCH_3_), 6.47 (2H, d, *J* = 8.5 Hz, 3″,5″-H), 6.64 (2H, d, *J* = 8.5 Hz, 3″,5″-H), 6.84 (1H, s, 6-H), 7.02 (1H, d, *J* = 3.5 Hz, 6′-H), 7.43 (1H, d, *J* = 7.5 Hz, 5′-H), 7.40–7.55 (2H, m, ArH), 7.61 (1H, d, *J* = 1.5 Hz, 7-H), 7.65 (1H, s, 2′-H), 8.00 (1H, d, *J* = 1.5 Hz, 9-H), 9.10 (1H, d, *J =* 3.0 Hz, 3-NH); ^13^C-NMR (125 MHz, DMSO-*d*_6_) 55.5, 69.9, 104.1, 113.6, 114.3, 115.5, 116.4 (d, ^2^*J*_CF_ = 21.9 Hz), 121.4, 126.6, 127.1, 127.6 (d, ^3^*J*_CF_ = 3.8 Hz), 128.4, 130.8 (d, ^3^*J*_CF_ = 8.5 Hz), 133.5, 137.1, 141.5, 159.7, 160.2, 162,7 (d, ^1^*J*_CF_ = 246.0 Hz); HRMS (ES): MH^+^, found 467.0139. C_23_H_17_N_2_O_2_^35^Cl^79^Br^+^ requires 467.0162.

### 3.5. Materials and Methods for Bioassays

#### 3.5.1. Materials and Methods for In Vitro Cytotoxicity Assay

##### Cell Culture

Human breast cancer (MCF-7) cells, mouse endothelial (End.2) cells derived from benign vascular tumors, and mouse melanoma (B16) cells were cultured in Dulbecco’s Minimum Essential Medium (DMEM) supplemented with 10% fetal calf serum (FCS), 1% penicillin-streptomycin and 20 mM glutamine. The cells were maintained in a 37 °C incubator in a humidified atmosphere containing 5% CO_2_.

##### Cell Viability Assay

Cell viability was assessed using the crystal violet nuclear staining assay. Cells were seeded in 96-well culture plates at a density of 5000 cells/well for 24 h, and then treated with test compounds (0–50 μM) or 0.05% dimethylsulfoxide (DMSO) for 48 h. The time and dose range were chosen following initial screening undertaken over a 72 h period. Following 48 h of treatment, the cells were fixed with 1% glutaraldehyde in phosphate buffered saline (PBS) for 15 min, and stained with a 0.1% crystal violet solution (Sigma-Aldrich, St. Louis, MO, USA). After 30 min the cells were incubated in 0.1% Triton X-100 (Sigma-Aldrich) for 90 min. The absorbance was read at 570 nm on an ELx 800 Universal Microplate Reader (Bio-Tek Instruments Inc., Analytical Diagnostic Products, Weltevreden, South Africa. Three wells were analysed for each concentration. The percentage of viable cells was calculated as follows: viability (%) = [A570 (treated) − A570 (blank)]/[A570 (control) − A570 (blank)] × 100 [22].

##### Statistics

The results are expressed as mean ± SD of at least three separate experiments. Data was analysed with GraphPad Prism 6.0 (GraphPad Software Inc., San Diego, CA, USA). One way analysis of variance (ANOVA) and post-hoc Tukeys test were used. Values of *p* < 0.05 were considered to be statistically significant.

#### 3.5.2. In Vitro pLDH Assay

Three-fold serial dilutions of the test compounds **4a**–**l** were incubated in triplicate with 3D7 strain *P. falciparum* parasites in a transparent 96-well flat bottom plate (Nest Biotechnology Co., Ltd., Wuxi, Jiangsu, China). DMSO and chloroquine were used as negative and positive controls, respectively. The plate was put in an airtight box, gassed and incubated with complete RPMI 1640 medium for 48 h. At the end of incubation, Malstat reagent was added to the 96-well plate followed by developing with NBT/PES (nitro blue tetrazolium + phenazine ethosulphate) reagent. Parasite growth was determined spectrophotometrically at 620 nm, by measuring the activity of the pLDH in control and drug-treated cultures using an Infinite F500 multiwell plate reader (Tecan Group Ltd., Männedorf, Switzerland). The OD values from control wells devoid of drug were referred to as having 100% pLDH activity. The IC_50_ are expressed as the % parasite survival relative to the control, calculated from fitted sigmoidal dose response curves. The dose response curves were obtained by plotting percentage parasite survival against the logarithm of the concentration using the GraphPad Prism software package. IC_50_ values were calculated graphically by interpolation from these curves. A prerequisite for all experiments was to have a Z′-factor > 0.5 as a measure of the quality of the screening assay.

## 4. Conclusions

In conclusion, we have demonstrated that the 2-amino-3-(arylalkynyl)-5-bromobenzamide framework represents an important synthon for the construction of novel 2,3-dihydro-1*H*-pyrrolo-[3,2,1-*ij*]quinazolin-1-ones via cyclocondensation and metal-assisted intramolecular C-N cyclization of the incipient 8-alkynylated quinazolinones. Hitherto, the preparation of the pyrroloquinazolinones and their quinazoline derivatives has generally been based on the construction of the quinazolinone or quinazoline moiety onto the 5-amino–substituted indole scaffold. A combination of the 3-(4-methoxyphenyl)- and 5-(4-fluorophenyl) groups in **4h** is desirable for cytotoxicity against the three cancer cell lines. The presence of 3-(4-fluorophenyl)- and 5-(3-chlorophenyl) groups in **4i**, on the other hand, resulted in significant cytotoxicity and selectivity against vascular tumor endothelial cells (End-2). The general lack of activity of the 2,3-dihydro-1*H-*pyrrolo[3,2,1-*ij*]quinazolin-1-ones against of *P. falciparum* (3D7), on the other hand, suggest that these compounds exhibit less or no binding affinity against lactate dehydrogenase (pLDH) and are therefore not worthy of further studies for antimalarial activity. Compounds **4h** and **4i** represent suitable candidates for further studies of biological activity to establish the origin of the observed cytoxicity and their mode of action.

## Figures and Tables

**Table 1:** IC_50_ values of **4a**–**k** expressed in μM against breast (MCF-7), melanoma (B-16) and endothelioma (sEnd.2) cells.

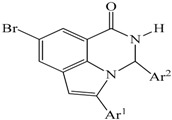					
4a–l					
			IC_50_ (μM)		
Compound	Ar^1^	Ar^2^	MCF-7	B-16	sEnd.2
**4a**	C_6_H_5_-	C_6_H_5_-	>100	>100	27.64
**4b**	C_6_H_5_-	4-FC_6_H_4_-	>100	>100	>100
**4c**	C_6_H_5_-	4-ClC_6_H_4_-	>100	>100	>100
**4d**	C_6_H_5_-	4-MeOC_6_H_4_-	37.10	>100	39.78
**4e**	4-FC_6_H_4_-	C_6_H_5_-	>100	39.61	32.27
**4f**	4-FC_6_H_4_-	4-FC_6_H_4_-	>100	>100	>100
**4g**	4-FC_6_H_4_-	4-ClC_6_H_4_-	>100	>100	>100
**4h**	4-FC_6_H_4_-	4-MeOC_6_H_4_-	0.83	0.66	0.95
**4i**	3-ClC_6_H_4_-	C_6_H_5_-	9.36	11.35	0.80
**4j**	3-ClC_6_H_4_-	4-FC_6_H_4_-	>100	>100	>100
**4k**	3-ClC_6_H_4_-	4-ClC_6_H_4_-	>100	>100	>100

**Table 2:** IC_50_ values of compounds **4a**–**l** and chloroquine against the 3D7 strain of *P. falciparum*.

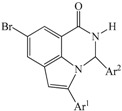			
4a–l			
Compound	Ar^1^	Ar^2^	IC_50_ (µM)
**4a**	C_6_H_5_-	C_6_H_5_-	32.18
**4b**	C_6_H_5_-	4-FC_6_H_4_-	43.31
**4c**	C_6_H_5_-	4-ClC_6_H_4_-	13.33
**4d**	C_6_H_5_-	4-MeOC_6_H_4_-	35.82
**4e**	4-FC_6_H_4_-	C_6_H_5_-	41.11
**4f**	4-FC_6_H_4_-	4-FC_6_H_4_-	37.15
**4g**	4-FC_6_H_4_-	4-ClC_6_H_4_-	15.51
**4h**	4-FC_6_H_4_-	4-MeOC_6_H_4_-	14.70
**4i**	3-ClC_6_H_4_-	C_6_H_5_-	64.56
**4j**	3-ClC_6_H_4_-	4-FC_6_H_4_-	12.65
**4k**	3-ClC_6_H_4_-	4-ClC_6_H_4_-	>100
**4l**	3-ClC_6_H_4_-	4-MeOC_6_H_4_-	11.45
**Chloroquine**			0.012

## Supplementary Materials

Supplementary materials can be accessed at: http://www.mdpi.com/1420-3049/22/1/55/s1.
