# Supplementary material for: Novel 2,3-Dihydro-1H-pyrrolo[3,2,1-ij]quinazolin-1-ones: Synthesis and Biological Evaluation
Source: Molecules. 2016 Dec 30;22(1):55. doi: 10.3390/molecules22010055 (PMC6155753; doi:10.3390/molecules22010055)
Supplement: Supplementary file 1 [file molecules-22-00055-s001.pdf]

Supplementary Materials: Novel 2,3-dihydro-1*H*-pyrrolo[3,2,1-*ij*]quinazolin-1-ones: Synthesis and Biological Evaluation

Malose J. Mphahlele, Tebogo A. Khoza and Peaceful Mabeta

Supplementary 1: Anticancer effect and IC_{50} values of compounds 4a–k S1–S8

Supplementary 2: % cell viability and IC_{50} values of chloroquine and compounds 4a–l S9–S15

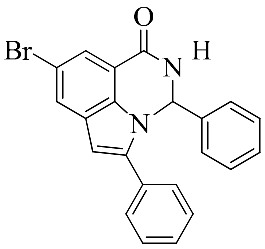

4a

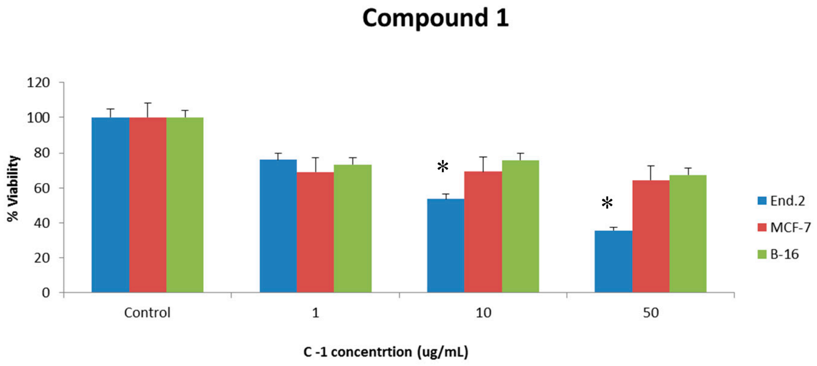

Figure S1. Anticancer effect of compound **4a**. * Significant activity at the indicated concentration.

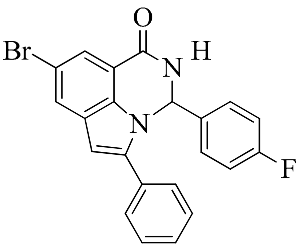

4b

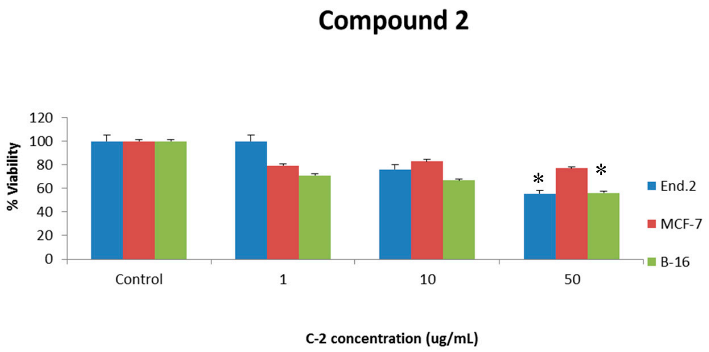

Figure S2. Anticancer effect of compound **4b**. * Significant activity at the indicated concentration.

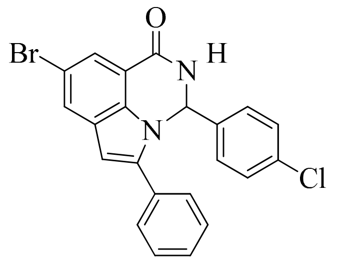

4c

Compound 3

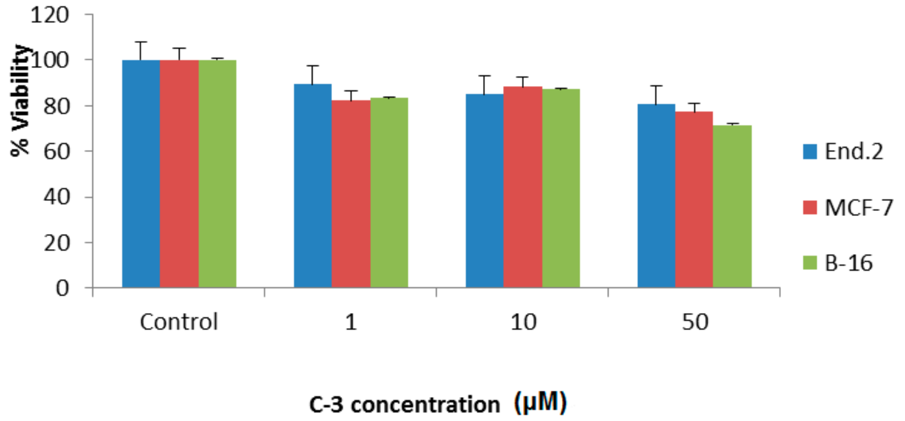

Figure S3. Anticancer effect of compound **4c**.

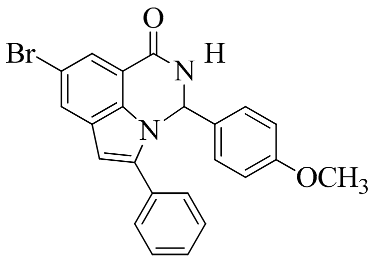

4d

Compound 4

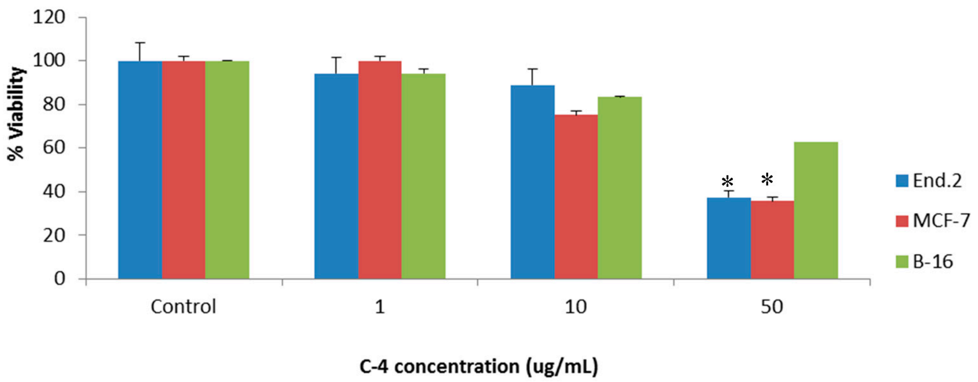

Figure S4. Anticancer effect of compound **4d**. * Significant activity at the indicated concentration.

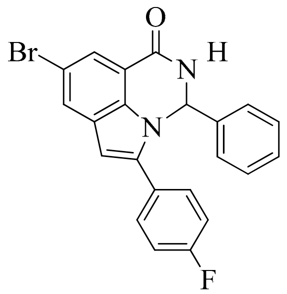

4e

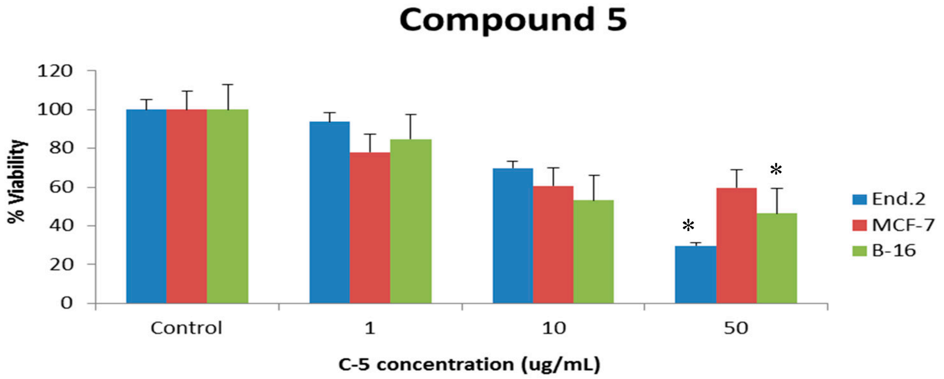

Figure S5. Anticancer effect of compound **4e**. * Significant activity at the indicated concentration.

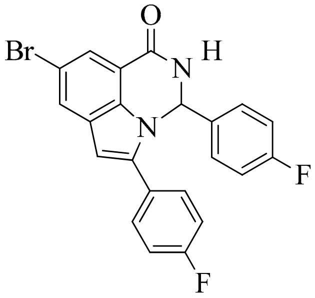

4f

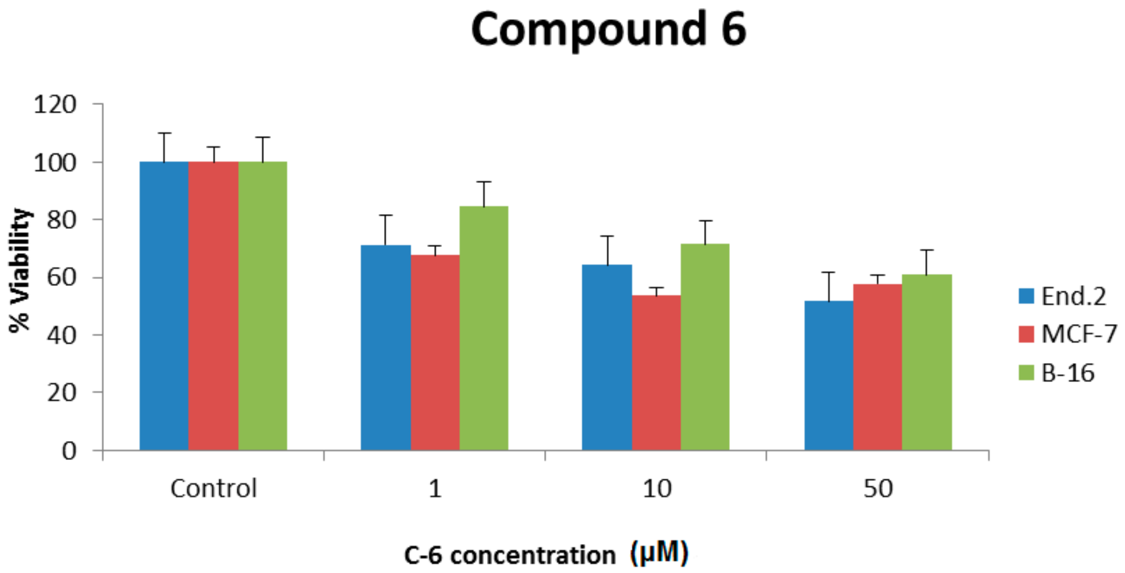

Figure S6. Anticancer effect of compound **4f**.

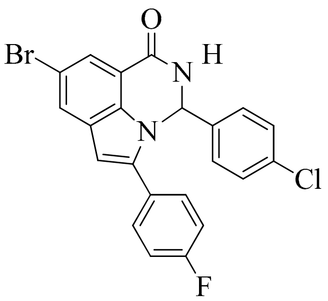

4g

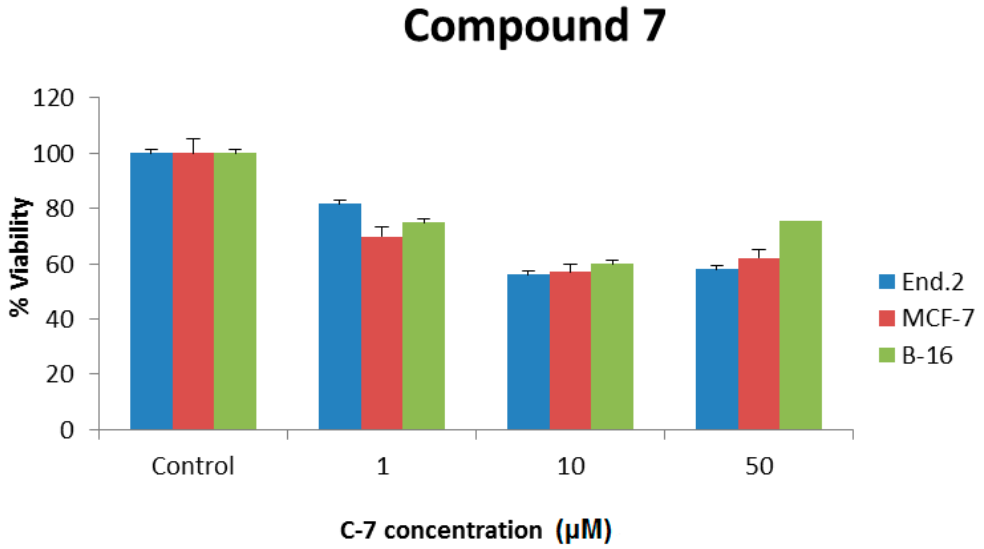

Figure S7. Anticancer effect of compound **4g**.

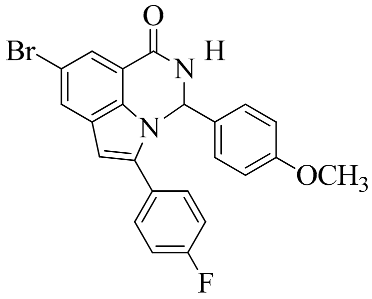

4h

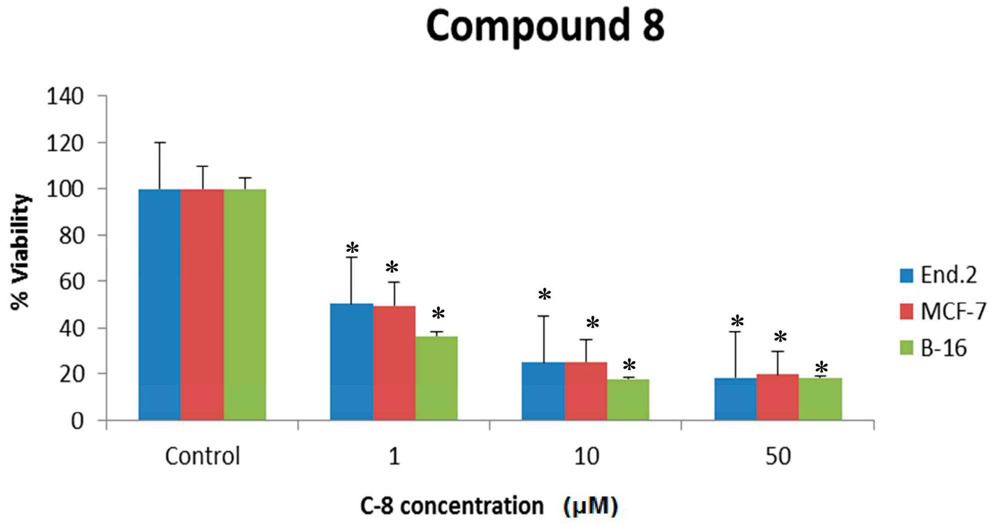

Figure S8. Anticancer effect of compound **4h**. * Significant activity at the indicated concentrations.

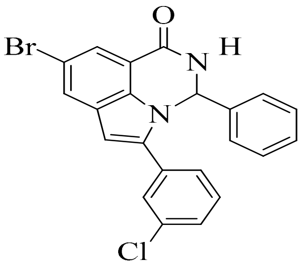

4i

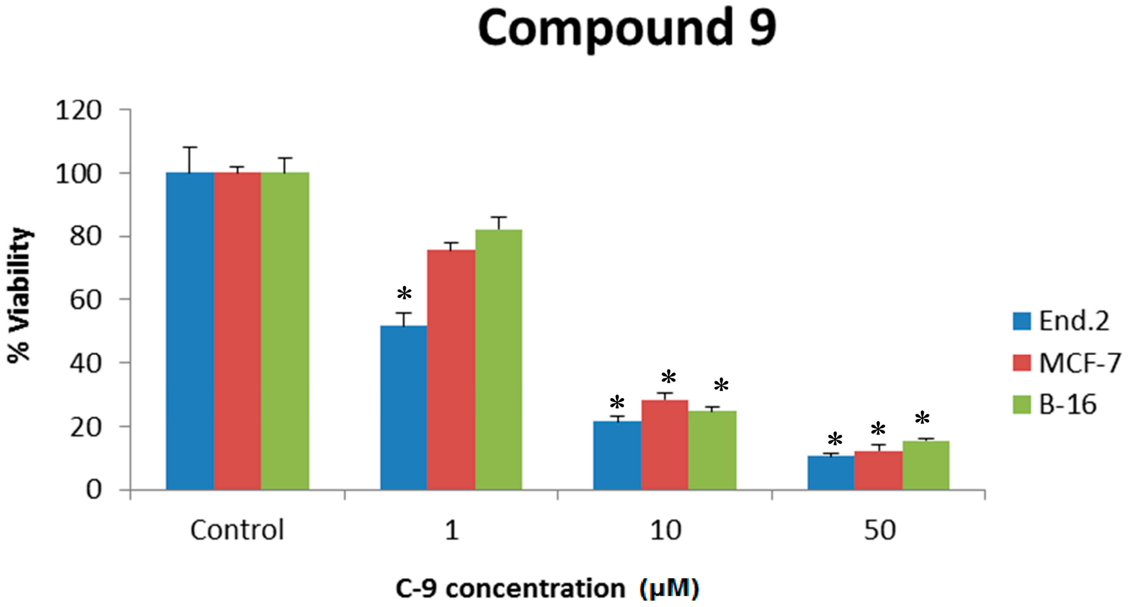

Figure S9. Anticancer effect of compound **4i**. * Significant activity at the indicated concentrations.

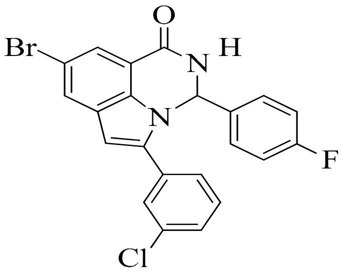

4j

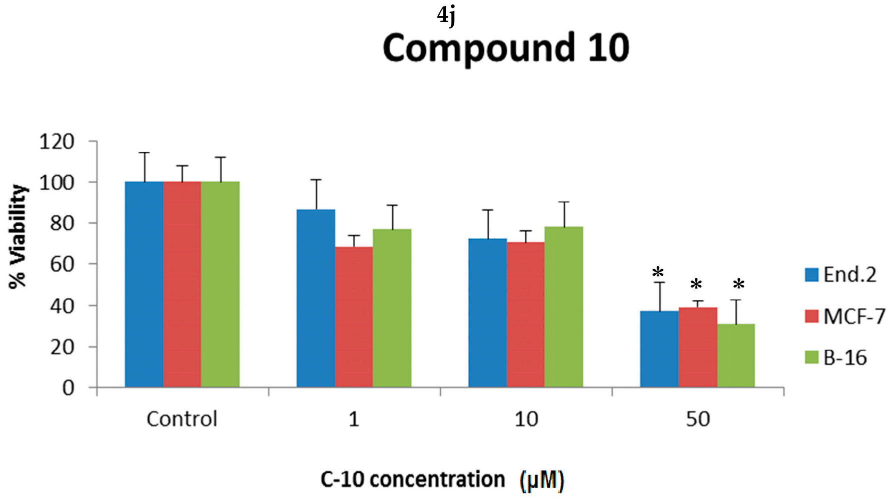

Figure S10. Anticancer effect of compound **4j**. * Significant activity at the indicated concentration.

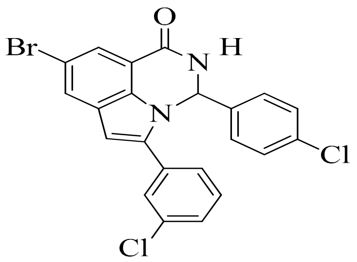

4k

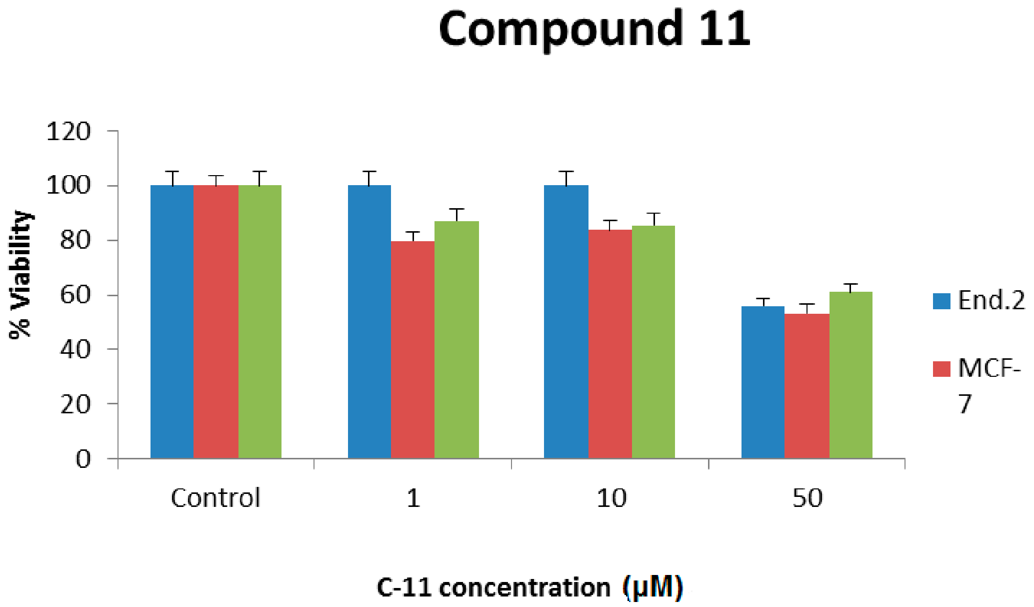

Figure S11. Anticancer effect of compound **4k**.

Table S1. IC_{50} values the concentration of compound that reduced cell viability by half.

Compound	Cell Line	IC_{50} Value
**4a**	MCF-7	-
	B16	-
	sEnd.2	27.6424
**4b**	MCF-7	-
	B16	-
	sEnd.2	-
**4c**	MCF-7	-
	B16	-
	sEnd.2	-
**4d**	MCF-7	37.0972
	B16	-
	sEnd.2	39.7852
**4e**	MCF-7	-
	B16	39.6101071
	sEnd.2	32.26803272
**4f**	MCF-7	-
	B16	-
	sEnd.2	-
**4g**	MCF-7	-
	B16	-
	sEnd.2	-
**4h**	MCF-7	0.8352
	B16	0.6602
	sEnd.2	0.951
**4i**	MCF-7	9.3632

	B16	11.3524
	sEnd.2	0.8021
	MCF-7	32.1029
**4j**	B16	34.6036
	sEnd.2	36.2328
	MCF-7	-
**4k**	B16	-
	sEnd.2	-

No IC$_{50}$ value, the compound did not induce 50% inhibition of cell growth at the doses tested.

Supplementary 2: % cell viability and LC$_{50}$ values of chloroquine and compounds 4a–l

Table S2. Log solvent percentage vs. % Parasite survival data used to plot dose-response curves for chloroquine.

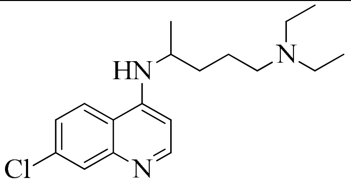

Chloroquine

Log Conc (nM) [a]	CQ	
	%PS	SD
3.000	−3.66	1.36
2.520	−3.75	2.73
2.040	−1.46	0.30
1.560	4.74	0.12
1.090	51.04	1.72
0.614	88.68	5.91
0.137	105.43	0.36
−0.340	103.66	3.89
−0.818	105.77	3.45
−1.294	100.93	3.46

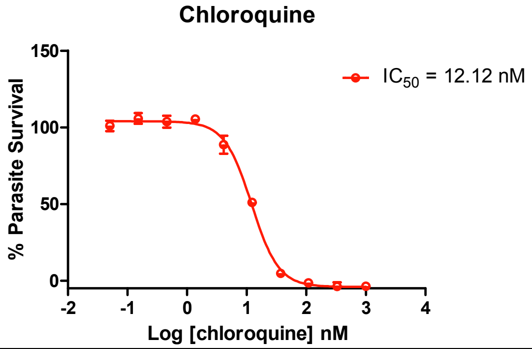

[a] Data are expressed as means of triplicate values. SD = standard deviation; %PS = Percentage Parasite Survival.

Table S3. Log concentration vs. % Parasite survival data used to plot dose-response curve for **4a**.

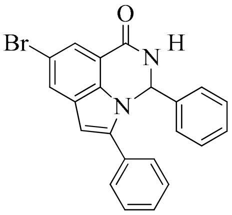

4a

Log Conc (μM) [a]	Tebo01	
	%PS	SD
2.00	19.92	3.80
1.52	56.77	1.27
1.04	97.04	4.47
0.57	103.71	2.79
0.09	102.54	4.87
−0.39	101.68	1.70
−0.86	99.90	1.93
−1.34	96.31	5.43
−1.82	96.31	4.08
−2.29	93.81	9.68

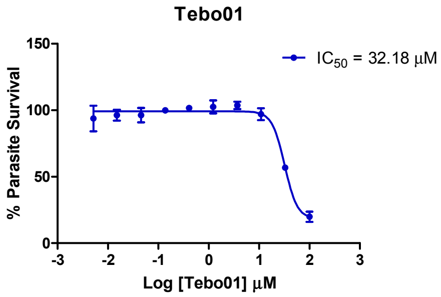

[a] Data are expressed as means of triplicate values. SD = standard deviation; %PS = Percentage Parasite Survival.

Table S4. Log concentration vs. % Parasite survival data used to plot dose-response curves for **4b**.

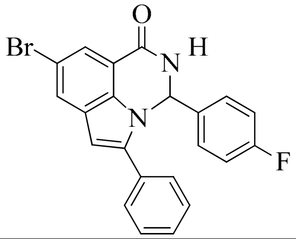

4b

Log Conc (μM) [a]	Tebo02 %PS	Tebo02 SD
2.00	14.24	4.30
1.52	52.02	2.07
1.04	87.32	3.19
0.57	95.05	3.90
0.09	102.10	7.59
−0.39	104.35	0.70
−0.86	100.01	3.18
−1.34	101.46	2.83
−1.82	103.21	2.12
−2.29	110.79	3.11

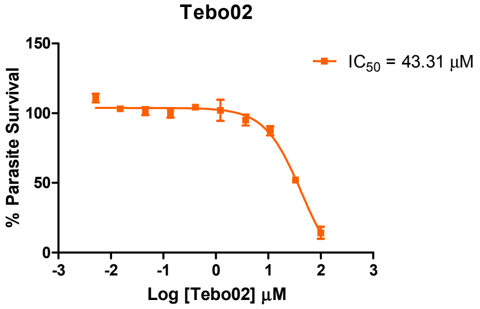

[a] Data are expressed as means of triplicate values. SD = standard deviation; %PS = Percentage Parasite Survival.

Table S5. Log concentration vs. % Parasite survival data used to plot dose-response curves for **4c**.

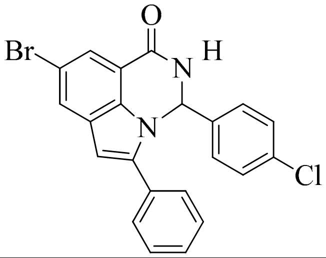

4c

Log Conc (nM) [a]	Teb03 %PS	Teb03 SD
2.00	8.37	3.63
1.52	14.73	4.15
1.04	63.70	5.40
0.57	87.29	3.51
0.09	94.62	4.03
−0.39	97.39	8.95
−0.86	96.46	1.75
−1.34	97.79	1.83
−1.82	99.26	6.91
−2.29	112.50	1.46

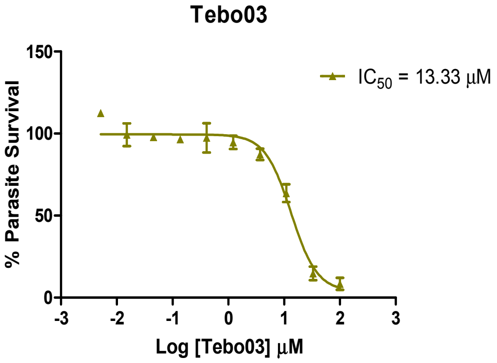

[a] Data are expressed as means of triplicate values. SD = standard deviation; %PS = Percentage Parasite Survival.

Table S6. Log concentration vs. % Parasite survival data used to plot dose-response curves for **4d**.

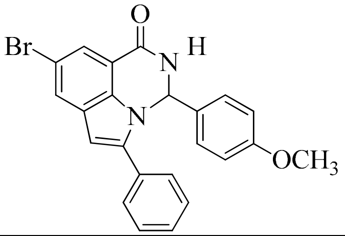

4d

Log Conc (nM) [a]	Tebo04	
	%PS	SD
2.00	26.99	4.05
1.52	69.73	2.14
1.04	105.61	1.56
0.57	113.35	3.79
0.09	109.42	7.12
−0.39	115.50	4.13
−0.86	104.23	3.60
−1.34	108.36	4.31
−1.82	105.60	1.09
−2.29	122.33	9.33

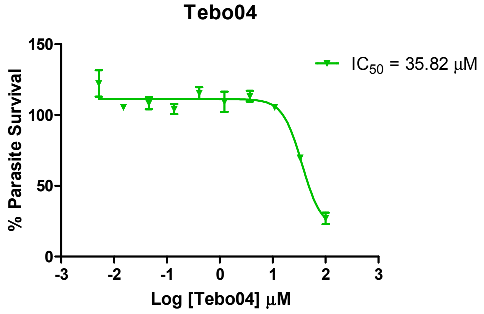

[a] Data are expressed as means of triplicate values. SD = standard deviation; %PS = Percentage Parasite Survival.

Table S7. Log concentration vs. % Parasite survival data used to plot dose-response curves for **4e**.

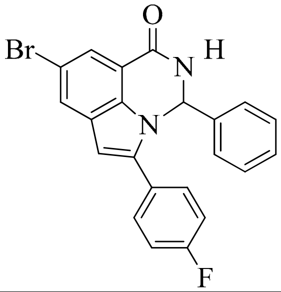

4e

Log Conc (nM) [a]	Tebo05	
	%PS	SD
2.00	16.37	5.27
1.52	57.56	1.79
1.04	96.11	3.30
0.57	109.73	2.55
0.09	103.84	4.34
−0.39	109.95	12.95
−0.86	112.05	2.95
−1.34	111.75	2.13
−1.82	110.16	2.98
−2.29	130.90	3.02

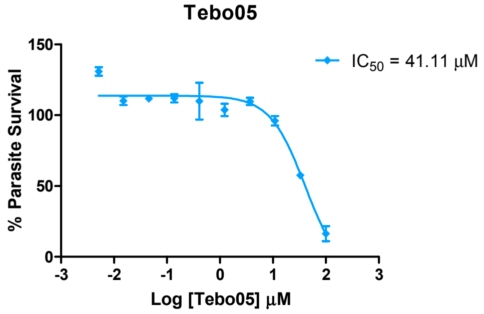

[a] Data are expressed as means of triplicate values. SD = standard deviation; %PS = Percentage Parasite Survival.

Table S8. Log concentration vs. % Parasite survival data used to plot dose-response curves for **4f**.

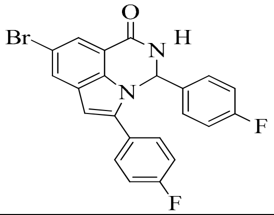

4f

Log Conc (nM)[a]	Tebo06	
	%PS	SD
2.00	8.25	1.00
1.52	61.83	5.11
1.04	90.74	7.44
0.57	97.14	2.39
0.09	104.34	5.43
−0.39	107.39	8.77
−0.86	110.36	8.83
−1.34	104.68	6.06
−1.82	110.13	6.94
−2.29	120.11	9.81

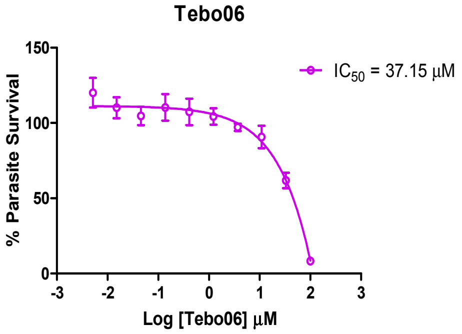

[a] Data are expressed as means of triplicate values. SD = standard deviation; %PS = Percentage Parasite Survival.

Table S9. Log concentration vs. % Parasite survival data used to plot dose-response curves for **4g**.

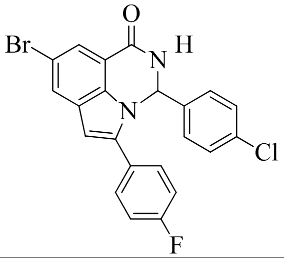

4g

Log Conc (nM) [a]	Tebo07	
	%PS	SD
2.00	7.38	2.43
1.52	21.05	1.55
1.04	65.00	2.82
0.57	86.48	4.67
0.09	94.07	1.87
−0.39	99.65	2.91
−0.86	106.60	8.92
−1.34	100.76	9.29
−1.82	105.66	11.03
−2.29	112.99	13.61

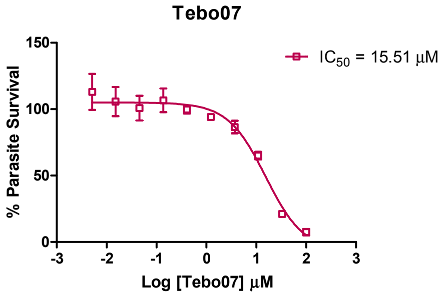

[a] Data are expressed as means of triplicate values. SD = standard deviation; %PS = Percentage Parasite Survival.

Table S10. Log concentration vs. % Parasite survival data used to plot dose-response curves for **4h**.

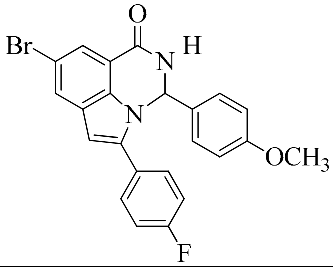

4h

Log Conc (nM) [a]	Tebo08	
	%PS	SD
2.00	11.18	3.23
1.52	20.60	2.73
1.04	72.16	3.27
0.57	94.94	1.63
0.09	100.22	4.63
−0.39	100.44	3.35
−0.86	97.98	3.36
−1.34	104.09	2.29
−1.82	104.19	2.28
−2.29	114.39	11.00

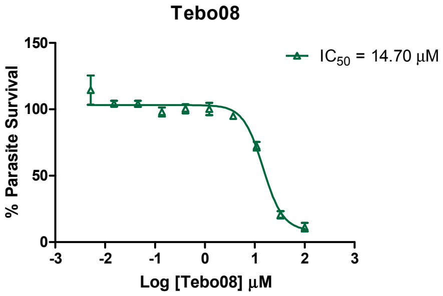

[a] Data are expressed as means of triplicate values. SD = standard deviation; %PS = Percentage Parasite Survival.

Table S11. Log concentration vs. % Parasite survival data used to plot dose-response curves for **4i**.

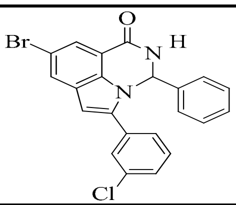

4i

Log Conc (nM) [a]	Tebo09	
	%PS	SD
2.00	28.87	6.33
1.52	79.55	2.41
1.04	99.04	5.52
0.57	97.83	1.88
0.09	101.15	7.50
−0.39	98.12	6.02
−0.86	94.40	5.96
−1.34	101.75	1.32
−1.82	103.29	4.03
−2.29	119.13	3.03

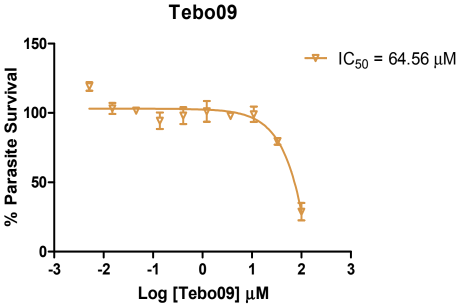

[a] Data are expressed as means of triplicate values. SD = standard deviation; %PS = Percentage Parasite Survival.

Table S12. Log concentration vs. % Parasite survival data used to plot dose-response curves.

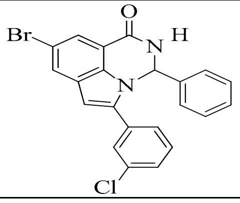

4j

Log Conc (nM) [a]	Tebo010	
	%PS	SD
2.00	11.24	5.79
1.52	7.33	6.00
1.04	69.63	0.71
0.57	95.08	8.57
0.09	106.25	8.18
−0.39	107.88	5.97
−0.86	107.93	8.21
−1.34	108.60	2.10
−1.82	109.83	3.36
−2.29	111.74	10.41

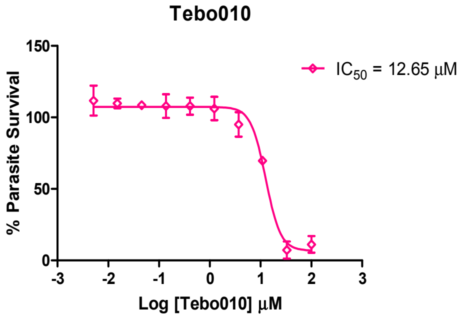

[a] Data are expressed as means of triplicate values. SD = standard deviation; %PS = Percentage Parasite Survival.

Table S12. Log concentration vs. % Parasite survival data used to plot dose-response curves.

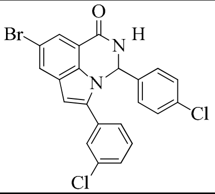

4k

Log Conc (nM) [a]	Tebo011	
	%PS	SD
2.00	66.16	4.30
1.52	99.20	9.26
1.04	93.16	3.65
0.57	111.33	2.49
0.09	102.76	4.66
−0.39	101.56	4.53
−0.86	105.41	3.55
−1.34	103.79	6.39
−1.82	112.91	3.13
−2.29	130.23	4.28

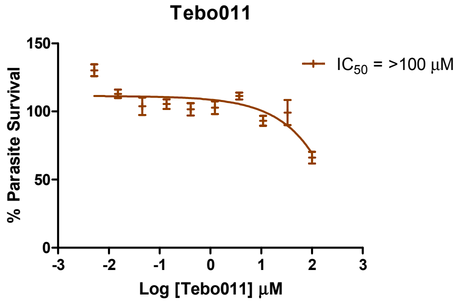

[a] Data are expressed as means of triplicate values. SD = standard deviation; %PS = Percentage Parasite Survival.

Table S13. Log concentration vs. % Parasite survival data used to plot dose-response curves.

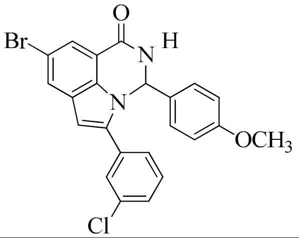

4l

Log Conc (nM) [a]	Tebo012	
	%PS	SD
2.00	18.08	1.79
1.52	20.90	3.34
1.04	64.67	4.87
0.57	93.85	7.80
0.09	97.75	10.95
−0.39	114.13	9.68
−0.86	108.23	13.16
−1.34	108.10	9.00
−1.82	98.40	8.75
−2.29	109.97	4.77

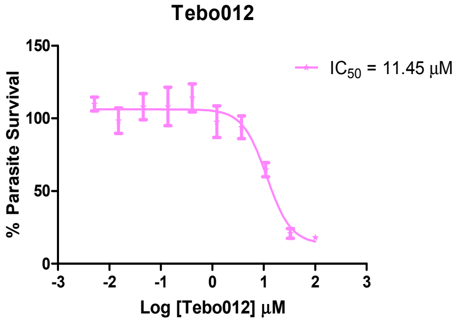

[a] Data are expressed as means of triplicate values. SD = standard deviation; %PS = Percentage Parasite Survival.
